# ﻿Taxonomic reassessment of chaetognaths (Chaetognatha, Sagittoidea, Aphragmophora) from Korean waters

**DOI:** 10.3897/zookeys.1106.80184

**Published:** 2022-06-21

**Authors:** Seohwi Choo, Man-Ki Jeong, Ho Young Soh

**Affiliations:** 1 Big data Fishery Resource Management Interdisciplinary Program, Chonnam University, Yeosu 59626, Republic of Korea; 2 Department of Smart Fisheries Resources Management, Chonnam National University, Yeosu 59626, Republic of Korea; 3 Department of Ocean Integrated Science, Chonnam National University, Yeosu 59626, Republic of Korea

**Keywords:** Arrow worms, chaetognaths, East China Sea, key, staining solution, taxonomy, voucher specimens

## Abstract

Since the first record of chaetognaths (arrow worms) reported from Korean waters by Molchanov in 1907, three families, 12 genera and 21 species have been additionally described. Eighteen of the 21 recorded species have been reported under scientific names different from the latest taxonomic system. This study aimed to address this issue by conducting a taxonomic re-evaluation of chaetognaths collected from Korean waters. Furthermore, the taxonomic usefulness of morphological differences in corona ciliata and distribution of ciliary sense receptors were re-examined using specimens stained with 1% Chlorazol black E (CBE) solution. This study includes taxonomically-validated voucher specimens of 18 species from Korean waters. Based on the specimens, re-description including image data and CBE staining pattern, distribution, ecological information and improved key were provided for each species. However, *Decipisagittadecipiens*, *Serratosagittaserratodentata* and *Sagittapseudoserratodentata* from Korean waters is still questioned because of the paucity of the voucher specimen and scientific literature.

## ﻿Introduction

The chaetognaths are marine mesoplanktonic carnivores present in most marine habitats and play an important role in the food web of pelagic ecosystems comprising connecting planktonic organisms of higher trophic levels. They have two sets of retractable chitinous grasping spines flanking a ventral mouth. They mostly feed on copepods, cladocerans, amphipods, krill and fish larvae depending on their size and developmental stage (Vega-Pérez 1995; Kruse et al. 2010). Occasionally, they also feed on organic debris (Grigor et al. 2020). These arrow-like creatures are of great ecological value, especially as a major food source for commercial fish, such as sardines and mackerel (Chacko 1949; Park 1970). Chaetognath species are distributed worldwide, including the Pacific, Atlantic, Indian and Antarctic Oceans. They are found in most of the vertical realms spanning from the surface to the bottom of the ocean (Müller et al. 2019; WoRMS 2022). As many species of arrow worm have different distributions depending on water mass, they have been historically used as research subjects to evaluate the marine environment (Russell 1936; Tokioka 1940; Bone et al. 1991; Terazaki 1992; Nagai et al. 2006). Their geographic distribution patterns have been used as important biological indicators to explain environmental physicochemical properties, such as cold current, warm current and oceanic frontal area (Park 1967; Park et al. 1990, 1991, 1992).

The phylum Chaetognatha was first mentioned as “arrow-shaped worms” by Slabber M (1769) and Krohn (1844) reported valuable anatomical features to characterise their internal organisation, nervous system, testes and chitinous cephalic armature. A comprehensive and detailed description of their morphology has been presented by Müller et al. (2019). The foundation of modern systematics of Chaetognatha was established by Ritter-Záhony (1911), who classified 27 species into six genera. Subsequently, the taxonomic categories from family to class were defined by Tokioka (1965a, b), who proposed an advanced classification system. He classified 58 species into two classes, two orders, five families and 15 genera. Of the two classes, Archisagittoidea comprises only fossil species, while Sagittoidea contains all the present chaetognaths existing today. The latter was subdivided into two orders, Phragmophora and Aphragmophora, based on the presence or absence of transversal musculature (i.e. phragms) in the body, respectively. Bieri (1991) proposed a comprehensive classification system for 114 species belonging to 22 genera and eight families. To date, the phylum Chaetognatha includes 133 species allocated to 26 genera and eight valid families (including Heterokrohniidae) (Müller et al. 2019). The taxonomic categories proposed by Tokioka (1965a, b) and Bieri (1991) are still mostly valid (WoRMS 2022), although recent molecular analyses have invalidated Pterosagittidae family (Gasmi et al. 2014; Nair et al. 2015; Müller et al. 2019; Peter et al. 2020).

Despite the long taxonomic history of Chaetognatha and its ecological importance, taxonomic research on Korean species is extremely limited. The first record of chaetognaths in Korean waters was presented by Molchanov (1907). Subsequently, Tokioka (1940) reported the geographical distribution of 13 species of order Aphragmophora. Including these 13 species, Park (1967, 1970, 1973) reported brief taxonomic and ecological features of 19 species. The number of chaetognath species in Korea was finally expanded to 21 by including two species described by Kim (1987). For the past 20 years, only ecological studies of chaetognaths in Korean waters have been carried out, based on these 21 species as indicators of various water masses and currents near the Korean Peninsula (Park et al. 1990, 1991, 1992; Yoo 1991; Yoo and Kim 1996, 1997; Nagai et al. 2006). The original descriptions and drawings by Park (1970) and Kim (1987) are the only studies available on Korean waters; however, both of them are theses for Doctoral and Master’s degrees, respectively, written only in Korean and are yet to be published. Furthermore, because none of the voucher specimens of 21 species used for description by Park (1970) and Kim (1987) are available and the accessible records of five species contain very short descriptions and sketches, it is difficult to confirm their presence in Korean waters. More importantly, these species have been reported under scientific names that are different from the latest taxonomic system. These taxonomic limitations regarding the Korean chaetognath taxa result in misidentification and low reliability of ecological research using indicator species.

Therefore, in this study, we aimed to accomplish the following: 1) to secure the first taxonomically verified voucher specimens of chaetognath from Korean waters and disclose them to public institutions; 2) to create the first comprehensive report of taxonomic features, including morphology, ecology and image information on Korean chaetognath species, based on newly-obtained voucher specimens; and 3) to provide an updated key to species for chaetognath taxa in Korea.

## ﻿Materials and methods

### ﻿Analysis of previous literature in Korea

**Figure 1.**
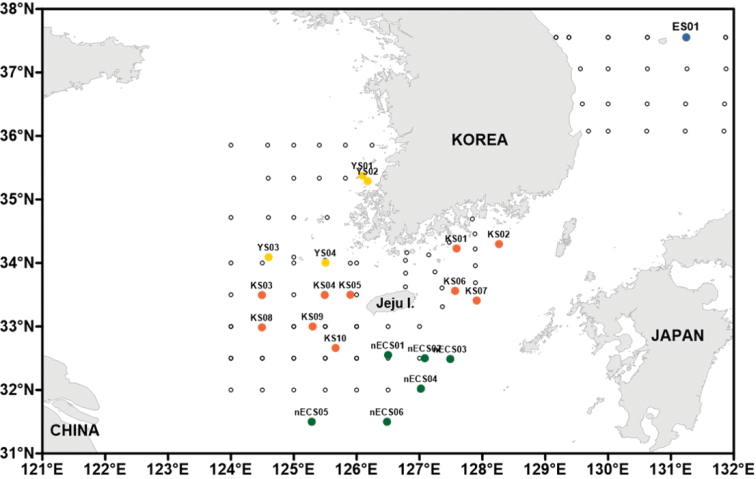
Sampling stations in Korean waters. Empty circles, sampling stations; filled circles, the stations where the chaetognaths were secured.

To understand the current status of Korean record on chaetognaths, a total of 14 taxonomic and ecological papers published since 1940 to date were investigated (Tokioka 1940, 1951; Park 1967, 1970, 1973; Park et al. 1990, 1991, 1992; Yoo 1991; Yoo and Kim 1996, 1997; Terazaki 1998; Nagai et al. 2006; Lee et al. 2016). The distribution of Korean chaetognath taxa mentioned in literature has been divided into four groups (the East Sea, Korea Strait, northern East China Sea and Yellow Sea) according to the physical characteristics of each sea near Korea (Fig. 1). All the mentioned species in literature (21 species of three genera, most belong to genus *Sagitta*) belong to order Aphragmophora, of which, three families, 12 genera and 21 species have been identified according to the traditional taxonomy conventions (Table 1). We performed a taxonomical comparative analysis of descriptions in previous literature and the newly-obtained specimens from Korean waters to confirm the existence of the 21 mentioned species in Korea, which were recorded in literature without voucher specimens.

**Table 1.** Korean chaetognath species list reported in previous studies. The species list consists of Tokioka (1940), Tokioka (1951), Park (1967), Park (1970), Park (1973), Park et al. (1990, 1992), Park et al. (1991), Yoo (1991), Yoo and Kim (1996), Terazaki (1998), Yoo and Kim (1997), Nagai et al. (2006). Abbreviation: ES = East Sea; KS = Korea strait; nECS = northern East China Sea; YS = Yellow Sea.

Taxa	ES	KS	nECS	YS	ES, KS, YS and nECS
Class Sagittidae					
Order Aphragmophora					
Family Krohnittidae					
**Genus *Krohnitta* Ritter-Zahony, 1910**					
*Krohnittapacifica* (Aida, 1897)	●	●		●	●
*Krohnittasubtilis* (Grassi, 1881)	●	●			●
Family Pterosagittidae (not valid)					
**Genus Pterosagitta Costa, 1869**					
*Pterosagittadraco* (Krohn, 1853)	●	●			●
Family Sagittidae					
**Genus *Aidanosagitta* Tokioka,1965**					
*Aidanosagittacrassa* (Tokioka, 1938)	●	●	●	●	●
*Aidanosagittaneglecta* (Aida, 1897)	●	●			●
*Aidanosagittaregularis* (Aida, 1897)	●	●		●	●
**Genus *Decipisagitta* Tokioka, 1965**					
*Decipisagittadecipiens* (Fowler, 1905)	●	●			●
**Genus *Ferosagitta* Kassatkina, 1971**					
*Ferosagittaferox* (Doncaster, 1902)	●	●			●
*Ferosagittarobusta* (Doncaster, 1902)	●	●			●
**Genus *Flaccisagitta* Tokioka, 1965**					
*Flaccisagittaenflata* (Grassi, 1881)	●	●	●	●	●
*Flaccisagittahexaptera* (D’Orbigny,1902)	●	●			●
**Genus *Mesosagitta* Tokioka, 1965**					
*Mesosagittaminima* (Grassi, 1881)	●	●			●
**Genus *Parasagitta* Tokioka, 1965**					
*Parasagittaelegans* (Verrill, 1873)	●	●	●		●
**Genus *Pseudosagitta* Germain & Joubin, 1912**					
*Pseudosagittalyra* (Krohn, 1853)	●	●			●
**Genus *Sagitta* Guoy & Gaimard, 1827**					
*Sagittabipunctata* Quoy & Gaimard, 1827	●	●			●
**Genus *Serratosagitta* Tokioka, 1965**					
*Serratosagittapacifica* (Tokioka, 1940)	●			●	●
*Serratosagittapseudoserratodentata* (Tokioka, 1940)	●	●			●
*Serratosagittaserratodentata*	●	●	●		●
**Genus *Zonosagitta* Tokioka, 1827**					
*Zonosagittabedoti* (Beraneck, 1895)	●	●	●		●
*Zonosagittapulchra* (Doncaster, 1902)		●			●
*Zonosagittanagae* Alvariño, 1967	●	●		●	●

### ﻿Morphological examination

**Figure 2.**
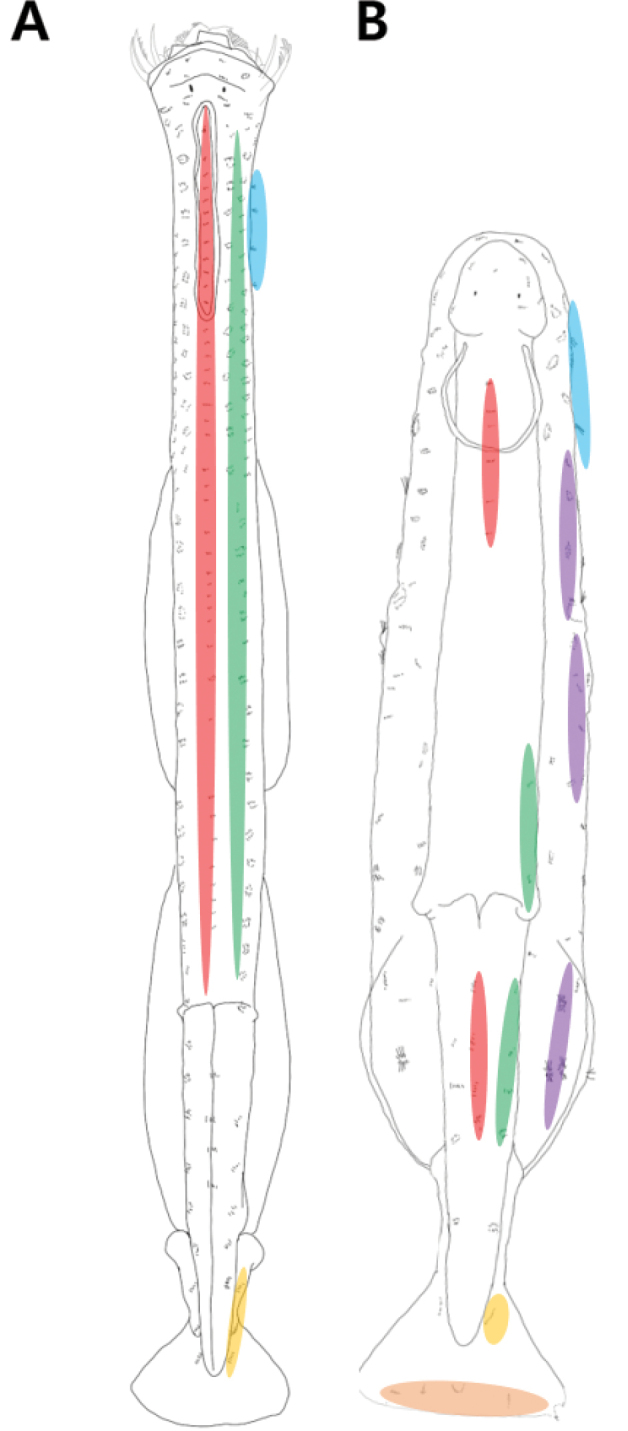
Schematic drawing showing the distribution of the ciliary sense organs (modified from Müller et al. 2014) **A***Ferosagittarobusta***B***Pterosagittadraco*. Colour indicated dorsomedian line (red); dorsolateral line (green); lateral line (blue); receptors on the lateral fin (purple); anterolateral receptors on the tail fin (yellow); posterior receptors on the tail fin (orange).

Field surveys were conducted at 20 stations in Korean waters from May 2019 to August 2020. Zooplankton collection was carried out from the bottom layer to the surface layer using a conical net (diameter: 0.6 m, mesh size: 200 μm) and MOCNESS (area of ​​mouth: 1 m^2^, mesh size: 200 μm). Samples were fixed with 5% formalin and the morphological features were observed using a stereo-optical microscope (DS-Fi3, Nikon, Japan). Furthermore, chaetognath specimens were identified at the species level by referring to the taxonomic terms suggested by Kapp (1991) and adults were isolated from the identified specimens according to Alvariño (1967). The quantitative and qualitative characteristics, based on Gasmi et al. (2014) (Table 2), were photographed using an optical light microscope equipped with a camera (DS-Fi3, Nikon, Japan) and analysed using the in-built software (NIS-Elements BR, version: 5.11.00, Nikon, Japan). Any features that were difficult to observe under the light microscope (including shape and location of corona ciliata rings, structure of fins and morphological patterns on the body surface) were confirmed by staining with Chlorazol black E (CBE) solution (1% in 95% ethanol and diluted in 3:7 ratio with distilled water prior to staining). The CBE pattern has been described as per Müller et al. (2014) (Fig. 2). To supplement the field specimens obtained in this study, 50 samples (eight genera, nine species) stored at the National Institute of Biological Resources (NIBR) were used for taxonomic re-examination. Based on the study by Kapp (1991), the taxonomic terminology and their abbreviations for chaetognath species identification used in this study are as follows: **AN**, anus; **AF**, anterior fin; **AT**, anterior teeth; **CC**, corona ciliata; **CF**, caudal fin; **COL**, collarette; **CL**, caudal length; **EP**, eye pigments; **GS**, grasping spine; **IN**, intestine; **ID**, intestinal diverticula; **LF**, lateral fin; **MO**, mouth; **OL**, ovary length; **O**, ovary; **PF**, posterior fin; **PT**, posterior teeth; **RLZ**, rayless zone; **SV**, seminal vesicle; **TL**, total length; **TM**, transverse muscle; and **VG**, ventral ganglion.

**Table 2.** Quantitative and qualitative characters of the chaetognaths used in this study (modified after Gasami et al. 2014).

	Quantitative characters		Qualitative characters
**C1**	Transverse muscles (absent/ present)	**Q1**	Total length (min/max)
**C2**	Body firmness (flaccid/ rigid)	**Q2**	Body/ tail ratio
**C3**	Body transparency (transparent, opaque, translucent)	**Q3**	Number of anterior teeth
**C4**	Collarette (absent/ present)	**Q4**	Number of posterior teeth
**C5**	Number of lateral fins (one pair/ two pairs)	**Q5**	Number of hooks (min/max)
**C6**	Fin positions. Anterior fins can be present on anterior part of ventral ganglion, middle of ventral ganglion, end of ventral ganglion, long distance behind the end of the ventral ganglion.		
**C7**	Comparison of anterior fins and posterior fins size		
**C8**	Rayless zone in the lateral fins (absent/ present)		
**C9**	Intestinal diverticula (absent/ present)		
**C10**	Type of hooks (gently curved/ gently curved and serrated/ abruptly curved)		
**C11**	Type of seminal vesicle (elongated, oval, spherical, conical)		
**C12**	Positions of seminal vesicle		
**C13**	Type of eye pigment (E, T, star, H, +, B shaped)		
**C14**	Type of corona ciliata (following Tokioka (1965))		
**C15**	Type of teeth row		
**C16**	Number of teeth row (only anterior row/ only posterior row/ presented both anterior and posterior)		

## ﻿Results

### ﻿General taxonomical characteristics

The size of the chaetognaths belonging to the order Aphragmophora ranged between 5 and 80 mm. Aphragmophora had no transverse muscles in the body. In general, species diagnosis in Aphragmophora was mostly based on the size and body appearance, the morphology of the intestine, the chitinous cephalic armature (shape and number of grasping spines and teeth) and the shape and position of the corona ciliata, lateral fins and seminal vesicles.

### ﻿Transparency of the body

**Figure 3.**
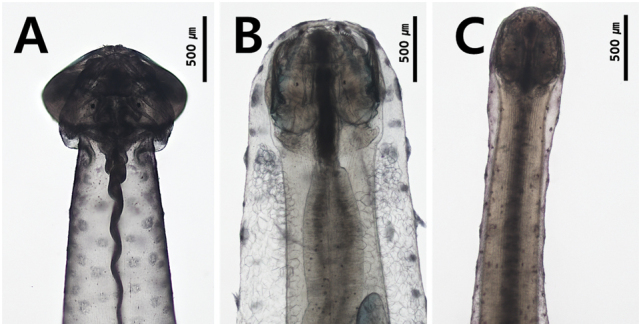
Appearance of body **A** transparent and flaccid body (*Flaccisagittaenflata*) **B** translucent body (*Pterosagittadraco*) **C** opaque and rigid body (*Aidanosagittacrassa*).

The transparency of the body is related to the development of longitudinal muscles in the trunk and tail and is a distinguishing characteristic at the generic level (Bieri 1991). However, the criteria for classifying the transparency of the body were not clear in previous studies. In this study, body transparency was classified into three types: first, the transparent type, which has weak and flexible muscles; second, the translucent type, where the internal organs can be observed from the dorsal side (e.g. digestive apparatus and ovaries), but the ventral ganglion is not visible; and third, the opaque type with strong, rigid muscles, with internal organs and ventral ganglion being invisible from the dorsal side. Representative genera of these categories were *Flaccisagitta* (Fig. 3A), *Pterosagitta* (Fig. 3B) and *Aidanosagitta* (Fig. 3C), respectively.

### ﻿Fins

**Figure 4.**
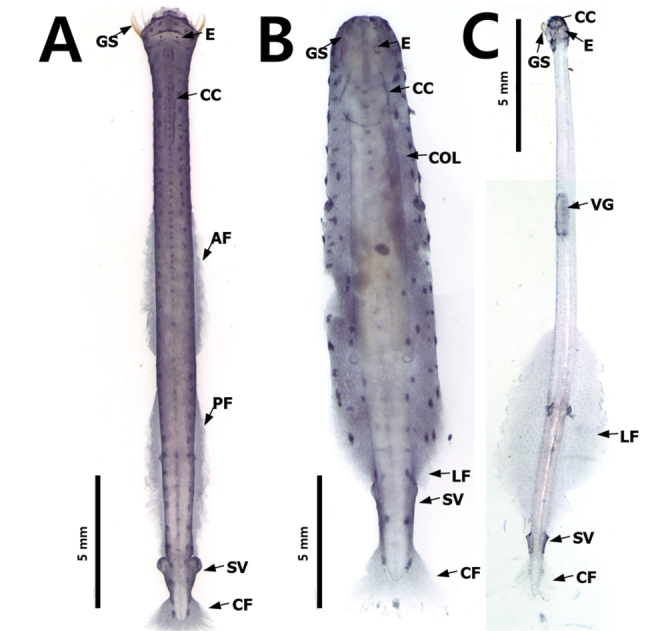
Three families inhabiting Korean waters **A***Ferosagittarobusta* (Sagittidae) **B***Pterosagittadraco*; **C***Krohnittasubtilis* (Krohnittidae). Abbreviations: AF = anterior fin; CC = corona ciliata; COL = collarette; CF = caudal fin; E = eye; GS = grasping spine; LF = lateral fin; PF = posterior fin; SV = seminal vesicle; VG = ventral ganglion.

The fins are used for floating and balancing (Hyman 1959). The parts of the body between the fins and the distribution of the fins on the body are morphological features of all the species (Duvert and Salat 1990); however, each species has its characteristic fin size and position. Although fins are easily damaged during the collection and fixation process, they are conspicuous characteristics of chaetognaths. They are located on the lateral and terminal parts of the body and their size, location and starting point are key characteristics. In this study, the number of fins was used as a feature to distinguish families; specimens with one pair of fins on the lateral sides of the body belonged to the families Krohnittidae and genus *Pterosagitta* and those with two pairs of fins belonged to families Sagittidae (Fig. 4A–C).

**Figure 5.**
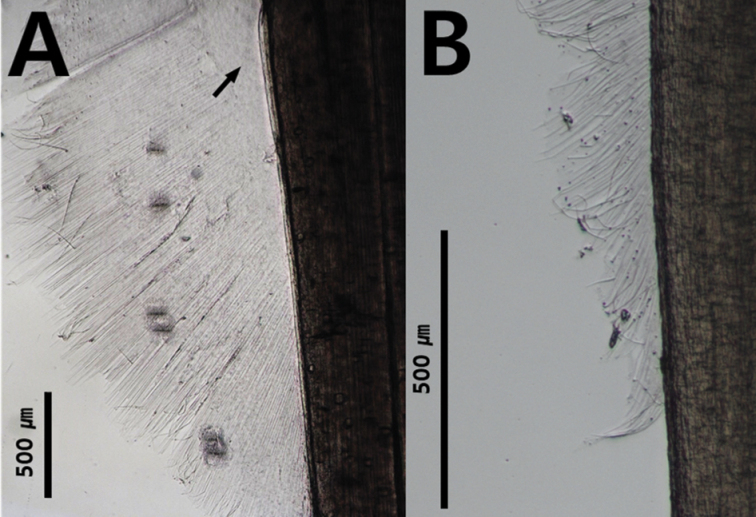
Presence and absence of rayless zone on lateral fins **A** black arrow shows rayless zone on posterior fin (*Zonosagittanagae*) **B** completely rayed fin (*Aidanosagittacrassa*).

Another diagnostic character at the genus and species level is the presence of a rayless zone in the lateral fins. For example, *Zonosagitta* has a long rayless zone on the anterior fins, but *Aidanosagitta* does not have a rayless zone on either anterior or posterior fins (Fig. 5A, B). The starting and ending points of the fins are also important taxonomic features. In general, the anterior fins begin at the anterior, middle or tip of the ventral ganglion. For instance, the anterior fins of *Pseudosagitta* and *Zonosagitta* begin at the anterior position to the ventral ganglion, while those of *Ferosagitta* and *Aidanosagitta* reach the ventral ganglion. On the contrary, the anterior fins of *Flaccisagitta* are located on the posterior part of the body far from the ventral ganglion.

### ﻿Seminal vesicles

**Figure 6.**
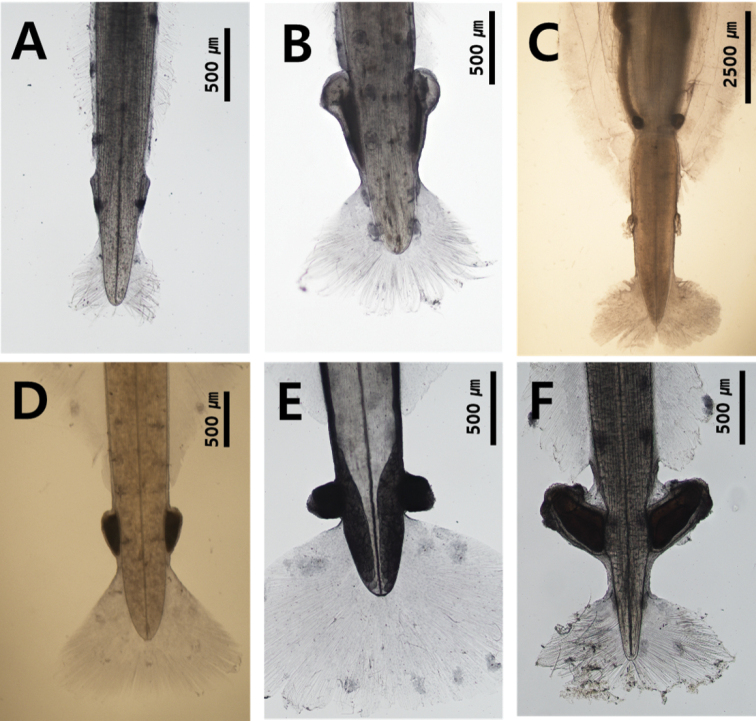
Shape and position of seminal vesicles **A***Aidanosagittaregularis* (elongated) **B***Ferosagittarobusta* (pear shape) **C***Pseudosagittalyra* (spherical shape) **D***Zonosagittanagae* (conical shape) **E***Flaccisagittaenflata* (spherical shape) **F***Serratosagittapacifica* (seminal shape with chitinous teeth).

All chaetognaths are hermaphroditic and have both female and male organs. In particular, the shape and location of seminal vesicles are distinct in different species. Seminal vesicles can be elongated along the lateral side of the tail (Fig. 6A) or have a pear, spherical or conical shape (Fig. 6B–E). *Serratosagittapacifica* has a distinct elongated knob with lateral protuberances. The chaetognath species can also be classified according to the location of seminal vesicles between the end of the posterior lateral fin and the caudal fin. The species can be classified, based on the vesicles that touch both posterior and caudal fins (Fig. 6A, D), those close to one of the two fins (Fig. 6B, E, F) and those well-separated from the two fins (Fig. 6C).

### ﻿Intestinal diverticula

**Figure 7.**
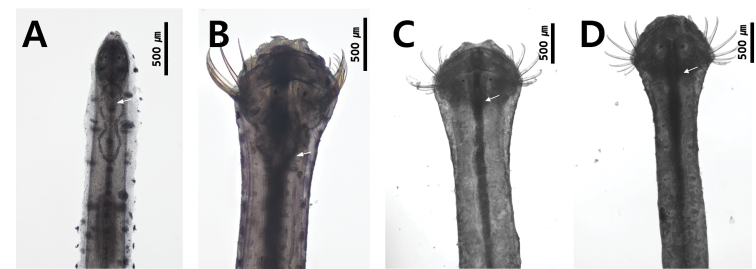
Intestinal diverticular (shown by white arrow) **A***Aidanosagittaregularis* (present) **B***Ferosagittarobusta* (present) **C***Zonosagittanagae* (absent) **D***Zonosagittabedoti* (absent).

The digestive apparatus of the chaetognath is in a single line from the mouth to the anus located just anterior to the posterior septum; the intestine extends in the trunk, but is not present in the tail. Classification can be done, based on the presence or absence of two intestinal diverticula located in the most anterior part of the intestine. They are clearly observed in the genera *Aidanosagitta* (Fig. 7A) and *Ferosagitta* (Fig. 7B), but not in *Pterosagitta* (Fig. 3B) and *Zonosagitta* (Fig. 7C, D).

### ﻿Grasping spines

**Figure 8.**
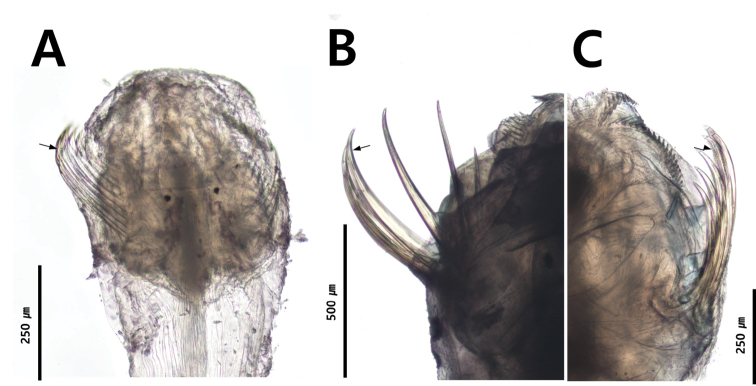
Grasping spine **A** abruptly curved hooks (*Krohnittasubtilis*, Krohnittidae) **B** gently curved and not serrated hooks (*Ferosagittarobusta*, Sagittidae) **C** gently curved and serrated hooks (*Serratosagittapacifica*, Sagittidae).

The grasping spines are laterally attached to the head of chaetognaths and are used for capturing and swallowing prey. The grasping spines of the family Krohnittidae are sharply curved, while those of Sagittidae are gently curved (Fig. 8A–C; Tokioka 1965a). The grasping spines of the genus *Serratosagitta* belonging to Sagittidae are serrated (Fig. 8C; Tokioka and Pathansali 1963).

### ﻿Anterior and posterior teeth

**Figure 9.**
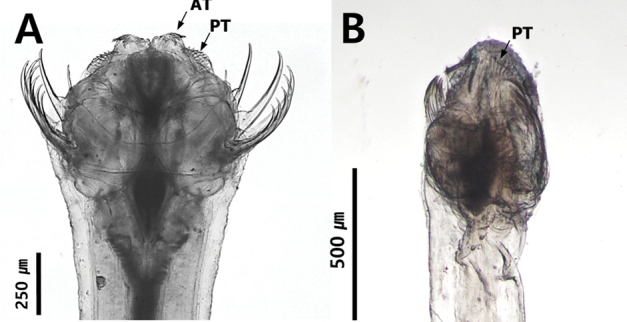
Number and shape of dentition **A***Ferosagittarobusta* (two rows) **B***Krohnittasubtilis* (one row).

The number of teeth rows is an important key to distinguish families (Tokioka 1965 a, b). Sagittidae (including *Pterosagittadraco*) has two rows of teeth arranged in a comb shape (Fig. 9A), while Krohnittidae has only one row of anterior teeth arranged in a fan shape (Fig. 9B).

### ﻿Corona ciliata

**Figure 10.**
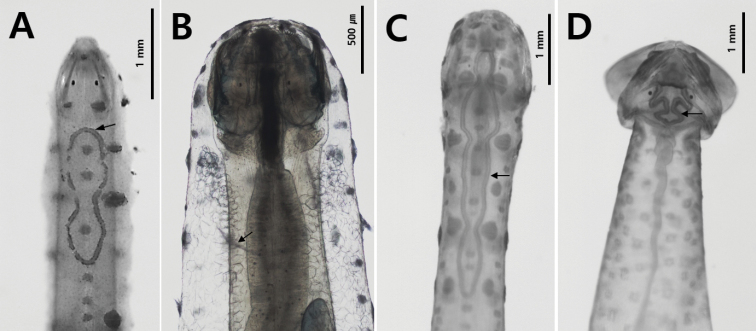
Position and shape of corona ciliata **A***Aidanosagittaregularis***B***Pterosagittadraco***C***Serratosagittapacifica***D***Flaccisagittaenflata*.

The corona ciliata is related to the sensory organs, presumably involved in chemoreception (Bleich et al. 2017) and is observed on the dorsal side of the specimen (Kapp 1991). It begins behind the eyes in *Aidanosagittaregularis* and *Pterosagittadraco* (Fig. 10A, B) or in front of the eyes in *Serratosagittapacifica* and *Flaccisagittaenflata* (Fig. 10C, D). The corona ciliata may also extend behind the neck, a short distance in the anterior trunk region (Fig. 10A, C) or does not exceed the head (Fig. 10D).

### ﻿Systematics

#### ﻿Order Aphragmophora

Taxon classificationAnimalia AphragmophoraKrohnittidae

Tokioka, 1965a

2E07F14A-1AA6-505A-B980-1875864BCF15

##### Diagnosis.

Ventral transverse musculature absent, less glandular structures on body surface. Grasping spines gently or abruptly curved (Tokioka 1965a). Collarette absent, present or small that is almost absent. Intestinal diverticula absent or present. One or two rows of teeth, teeth-rows arranged in comb or fan shape. One paired or two paired lateral fins with or without rayless zone (Alvariño 1967).

### ﻿Key to family of Aphragmophora

**Table:** 

1	One pair of lateral fins	**2**
–	Two pairs of lateral fins	**4**
2	One row of teeth, collarette absent	**Krohnittidae (present in Korea)**
–	Two rows of teeth	**3**
3	One pair of lateral fins on the tail, Collarette remarkably thick	***Pterosagittadraco* (present in Korea)**
–	One pair of lateral fins on the trunk	**Pterokrohniidae**
4	Two rows of teeth, neck contraction	**Bathybelidae**
–	Two rows of teeth	**Sagittidae (present in Korea)**

#### ﻿Family Krohnittidae

Taxon classificationAnimalia AphragmophoraKrohnittidae

Tokioka, 1965

55D706B0-F566-5BB6-AE1F-A77EBEF1384A

##### Diagnosis.

Small head. Grasping spines abruptly curved. One row of teeth. Collarette either short or absent. One pair of lateral fins arranged on the posterior trunk and tail.

#### ﻿Genus Krohnitta

Taxon classificationAnimalia AphragmophoraKrohnittidae

Ritter-Záhony, 1910

01E579D6-87C5-5A36-8FCC-AEF21187CF9F

##### Diagnosis.

Slender and transparent body. Lateral fins on the body and tail with rayless zone or partially rayed. Intestinal diverticula absent. Seminal vesicles oval or elongated touching both lateral fins and caudal fins.

### ﻿Key to species of *Krohnitta*

**Table:** 

1	One pair of fins with rayless zone. Seminal vesicles oval shaped and elongated, touching both paired fins and caudal fins	***K.subtilis***
–	One pair of rayed fins. Seminal vesicles oval shaped and placed dorsally at the point where lateral fins meet caudal fins	***K.pacifica***

#### ﻿ Krohnitta subtilis

Taxon classificationAnimalia AphragmophoraKrohnittidae

(Grassi, 1881)

927E8AAA-A4EE-5091-9FB5-68AAD13A5FD5

**Figure 11.**
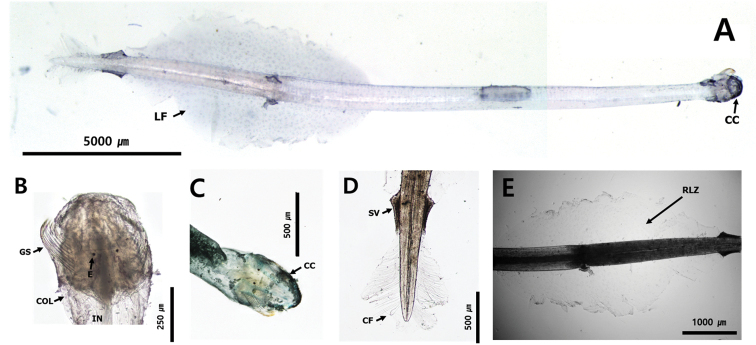
**A***Krohnittasubtilis* (dorsal view) **B** head **C** tail **D** lateral fin. Abbreviations: CC = corona ciliata; CF = caudal fin; COL = collarette; E = eye; GS = grasping spine; IN = intestine; LF = lateral fin; RLZ = Ray less zone; SV = seminal vesicle.

Figs 3C , 4C , 8A , 9B , 11A–D

Spadella subtilis : Grassi, 1883: 16 p., table 1.
Krohnia subtilis : Fowler, 1906: 25–26 p., figs 86–88; Michael 1908: 269–270 p.
Krohnitta subtilis : Burfield & Harvey, 1926: 117 p., figs 45–50.; Thomson 1947: 22 p.; Tokioka 1965: 352–353 p.; Alvariño 1967: 18–20 p., fig. 9 A–D; Srinivasan 1979: 37–39 p., fig. 21 A–D; Michel 1984: 30 p., fig. 41; McLelland 1989: 158 p., fig. 5A–D; Park et al. 1990: 74–76 p., fig. 52.; Nair et al. 2008: 210 p., table 1.

##### Material examined.

Korea Strait (33°30.000'N, 125°54.000'E), 0–90 m depth, oblique towing with conical net, Feb 2020, NIBRIV0000895313 (one specimen); northern East China Sea (33°00.000'N, 127°4.098'E), 0–110 m depth, oblique towing with conical net, Feb 2020, NIBRIV0000895312 (one specimen).

##### Description.

Total body length ranged between 10.8 and 11.5 mm and tail 27.3–33.7% of body length. Hooks 8–10. Anterior teeth 14. Slender and transparent body (Fig. 11A). Small head. One row of stout teeth arranged in fan shape (Fig. 9B). Collarette and intestinal diverticula absent (Fig. 11B). Grasping spine abruptly curved (Fig. 8A). Round eyes with eye pigments in “E” shape (Fig. 11B). Corona ciliata beginning in front of eyes with round shape (Fig. 11C). Lateral fins 29.4% of body length. Starting points of lateral fins 54.3% and ending points of lateral fins 82.2% of body length, respectively. One pair of lateral fins only rayed on outer edge, with forward ends equidistant from caudal septum (Fig. 11A, E). Caudal fin roughly round in shape (Fig. 11D). Seminal vesicles elongated with anterolateral-edge-opening touching both lateral fins and caudal fin (Fig. 11D).

##### Distribution.

This species is found in the epipelagic (0–200 m depth) and mesopelagic zones (200–500 m depth) of the Pacific, Indian and Atlantic Oceans (Pierrot-Bults and Nair 1991), the Indian water (George 1952) and the Tosa Bay in Japan (Ohnishi et al. 2014), while in this study, it was found in the epipelagic zone (0–110 m depth) of the Korea Strait and northern East China Sea (Fig. 1: stations KS05 and nECS04).

##### Ecology.

This cosmopolitan species can be found in tropical to temperate waters (Alvariño 1967). The temperature ranged between 16.40 and 16.41 °C and salinity was 34.58 psu at the sampling stations of this study.

##### Remarks.

This species is clearly distinguished from *K.pacifica* by the presence of a rayless zone in the lateral fin. Furthermore, the presence of a pair of lateral fins with a wide rayless zone and a fan-shaped dentition in *K.subtilis* collected from Korean waters are consistent with the records of Alvariño (1967) and Bieri (1991). However, the location of the corona ciliata (in front of the eyes, Fig. 11C) of the species found in Korean waters was different from that of the previous records (located behind the eye). No specific pattern was observed through CBE staining on the body surface.

#### ﻿ Krohnitta pacifica

Taxon classificationAnimalia AphragmophoraKrohnittidae

(Aida, 1897)

258F0961-6F86-5798-A600-51002241F0BA

Krohnitta pacifica : Tokioka, 1965: 352–353 p.; Alvariño 1967: 15–17 p., fig. 7A–E; Michel 1984: 29 p., fig. 40; Kim 1987: 35–36 p., plate 13; Park et al. 1990: 73 p., fig. 51.

##### Material examined.

Northern East China Sea (32°29.420'N, 127°29.654' E), 20–100 m depth, oblique towing with MOCNESS, Aug 2020 (one specimen).

##### Description.

Slender and transparent body. Small head. Grasping spines abruptly curved. One row of stout teeth arranged in fan shape. One pair of lateral fins partially rayed, forward and equidistant from caudal septum. Lateral fins positioned at anterior end, at level of caudal septum with rayed lateral fins. Caudal fin damaged. Collarette and intestinal diverticula absent. No seminal vesicle visible.

##### Distribution.

This species is found in the epipelagic zone (0–200 m depth) of the northern Indian Ocean (Pierrot-Bults and Nair 1991), the Gulf of Mexico (Pierce 1951) and the Japanese coast (Tosa Bay, Sagami and Suruga) (Nagasawa and Marumo 1972; Ohnishi et al. 2014) and, in this study, it was found in the epipelagic zone (20–100 m depth) of the northern East China Sea (Fig. 1: station nECS03).

##### Ecology.

An inhabitant of the surface layer of the warm oceanic waters (Tokioka 1940). Mature specimen was reported to be 6–8 mm in length (Alvariño 1967). The temperature range in the sampling stations of this study was 18.49–28.84 °C and salinity range was 30.71–34.59 psu.

##### Remarks.

Only immature individuals could be collected in this study. The specimens of *K.pacifica* we observed had one pair of fins and the structure of the fin was rayed, except for the base part close to the body. These were distinct characteristics of *K.pacifica* that differentiated it from *K.subtilis*. Since seminal vesicles were not observed in all observed Korean specimens, they were classified as immature stage (Alvariño 1967).

#### ﻿Family Pterosagittidae

Taxon classificationAnimalia AphragmophoraPterosagittidae

Tokioka, 1965a

828F54D9-E3CE-5E05-A607-698611FA857F

##### Diagnosis.

Wide head. Two rows of teeth. Collarette wide and extending through full body. One pair of rayed lateral fins located on the tail. Intestinal diverticula absent. Only one genus has been described within this family: *Pterosagitta* (Costa, 1869).

##### Remarks.

A previous phylogenetic study reported that Pterosagittidae is genetically quite close to Sagittdae (Gasmi et al. 2014; Nair et al. 2015; Peter et al. 2020; Müller et al. 2019). In this study, a recent research paper was reviewed and Pterosagittidae was marked as invalid (Table 1).

### ﻿Key to the species of *Pterosagitta*

**Table:** 

1	One pair of rayed lateral fins on the tail. Collarette extremely thick and extends from head to seminal vesicles. Seminal vesicles oval-shaped and elongated, touching both lateral fins and caudal fin	***P.draco***

#### ﻿ Pterosagitta draco

Taxon classificationAnimalia AphragmophoraPterosagittidae

(Krohn, 1853)

8AD65EE5-7DA2-5B6B-B749-64AABD5A4383

**Figure 12.**
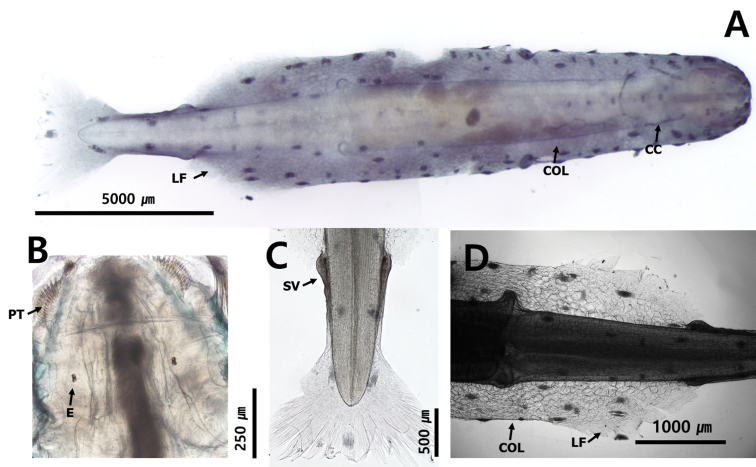
**A***Pterosagittadraco* (dorsal view) **B** head **C** tail **D** lateral fin. Abbreviations: CC = corona ciliata; COL = collarette; E = eye; LF = lateral fin; PT = posterior teeth; SV = seminal vesicle.

Figs 3B , 4B , 10B and 12A–D

Spadella draco : Grassi, 1883: 15 p.; Beraneck 1895: 154 p.; Aida 1897: 20 p., fig. 12; Doncaster 1902: 214–215 p.
Pterosagitta draco : Michael, 1919: 264–265 p., table 18; Thomson 1947: 22–23 p.; Tokioka 1965: 351–352 p.; Alvariño 1967: 21–22 p., fig. 11A–D; Srinivasan 1979: 34–35 p., fig. 19A–E; Michel 1984: 29 p., fig. 5; Kim 1987: 33–34 p., plate 12; Park et al. 1990: 71–73 p., fig. 50.

##### Material examined.

Northern East China Sea (32°30.000'N, 127°5.100'E), 0–120 m depth, oblique towing with conical net, Feb 2020, NIBRIV0000895299 (one specimen).

##### Description.

Total body length ranged between 6.5 and 9.1 mm and tail 38.4–40.1% of body length. Hooks 8. Anterior teeth 10 and posterior teeth 12. Rigid and translucent body (Fig. 12). Wide and angular head (Fig. 12). Wide collarette extending over entire body and reaching anterior of seminal vesicles (Fig. 12A. C). Rectangular eyes with “T” shaped eye pigments (Fig. 12B). Intestinal diverticula absent (Fig. 3B). Lateral fins 20.7% of body length. Starting point of lateral fins 65.2% and ending points of lateral fins 86.3% of body length, respectively. One pair of lateral fins triangular-shaped and completely rayed, with forward ends at level of caudal septum (Fig. 12A, D). Seminal vesicles posteriorly elongated with anterior knob touching lateral fins (Fig. 12C–D). Eggs reaching middle of body. Corona ciliata anteriorly opened with horseshoe shape beginning in neck region and ending just behind eyes (Figs 10B, 12A).

##### Distribution.

This species is located in the epipelagic (0–200 m depth) and mesopelagic zones (200–500 m depth) of the Pacific, Indian and Atlantic Oceans (Pierrot-Bults and Nair 1991), the epipelagic zone (0–200 m depth) of the Caribbean Sea (Michel,1984), the coastal waters surrounding India (George 1952) and the Tosa Bay in Japan (Ohnishi et al. 2014). In this study, it was found in the epipelagic zone (0–120 m depth) of the northern East China Sea (Fig. 1: station nECS02).

##### Ecology.

This species is widely distributed in warm water masses and appears all year round in Korean waters, except in the Yellow Sea (Kim 1987). The temperature range of the Caribbean Sea was reported as 22–29 °C, and salinity range was 33–38 psu (Michel 1984). At the sampling stations of this study, the temperature ranged between 15.83–28.80 °C and salinity ranged between 31.38–34.60 psu.

##### Remarks.

The largest specimen collected in this study was 9.1 mm in length, which was at stage 4 maturity. It was smaller than the specimen from New Zealand (16 mm) reported by Lutschinger (1993). It had characteristics consistent with the *Pterosagittadraco* reported from the Pacific (Alvariño 1967; Michel 1984), such as the presence of two rows of teeth, one pair of lateral fins, a corona ciliata located between the back of the eye and the neck and a broad collarette extending from the head to seminal vesicles. We observed one specimen for CBE staining pattern: dorsomedian line, 8 dots; dorsolateral line, 25 dots; lateral line, 10 dots; receptors on the lateral fin, 2 dots; anterolateral receptors on the caudal fin, 3 dots; posterior receptors on the caudal fin, 6 dots.

#### ﻿Family Sagittidae

Taxon classificationAnimalia AphragmophoraSagittidae

(Claus & Grobben, 1905)

E96B01F3-B94E-5A2A-891A-4106B46C513C

##### Diagnosis.

Two rows of stout teeth arranged in comb shape. Two pairs of lateral fins on the trunk and tail.

### ﻿Key to genus of Sagittidae

**Table:** 

1	Serrated grasping spines	***Serratosagitta* (present in Korea)**
–	Non-serrated grasping spines	**2**
2	Flaccid body	**3**
–	Rigid body	**6**
3	Intestinal diverticula present	**4**
–	Intestinal diverticula absent	**5**
4	Anterior fins beginning at the middle of ventral ganglion, the seminal vesicle closer to lateral fins than caudal fin	***Decipisagitta***
–	Anterior fins beginning between ventral ganglion and caudal septum, the seminal vesicle well-separated from lateral fins, but touching caudal fins	***Mesosagitta* (present in Korea)**
5	Anterior fins beginning far behind the end of the ventral ganglion	***Flaccisagitta* (present in Korea)**
–	Anterior fins beginning at the end of ventral ganglion	***Pseudosagitta* (present in Korea)**
6	Intestinal diverticula present	**7**
–	Intestinal diverticula absent	**9**
7	Collarette present	**8**
–	Collarette and rayless zone on lateral fins absent	***Parasagitta* (present in Korea)**
8	The head width greater than body width, short collarette, the rayless zone on lateral fins present, the corona ciliata beginning before the eye	***Ferosagitta* (present in Korea)**
–	The head width smaller than body width, well-developed collarette, completely rayed lateral fins, the corona ciliata beginning behind the eye	***Aidanosagitta* (present in Korea)**
9	Anterior part of lateral fins with rayless zone	***Zonosagitta* (present in Korea)**
–	Rayless zone on lateral fins absent	***Sagitta* (present in Korea)**

#### ﻿Genus Serratosagitta

Taxon classificationAnimalia AphragmophoraSagittidae

Tokioka & Pathansali, 1963

A32E314E-E859-5F28-9CF1-51DCB1270A7D

##### Diagnosis.

Intestinal diverticula either absent or present. Grasping spines serrated. Two rows of teeth. Two pairs of lateral fins either with rayless zone or completely rayed.

### ﻿Key to species of *Serratosagitta*

**Table:** 

1	Intestinal diverticula absent	**2**
–	Intestinal diverticula present	**3**
2	Seminal vesicles with forward elongated knob and teeth-like appendages forming 5–10 distal protrusions anteriorly, well-separated from caudal fin but touching posterior fins, the posterior fins and anterior fins of almost same length	***S.pacifica***
–	Seminal vesicles touching the posterior fins and caudal fin, the posterior fins longer than anterior fins	***S.serratodentata***
3	Seminal vesicles well-separated from caudal fin, but touching posterior fins, posterior fins and anterior fins of almost same length	***S.pseudoserratodentata***

#### ﻿ Serratosagitta pacifica

Taxon classificationAnimalia AphragmophoraSagittidae

(Tokioka, 1940)

A8ABB4FE-C60B-57B2-A94D-C4E8BD18CAE4

**Figure 13.**
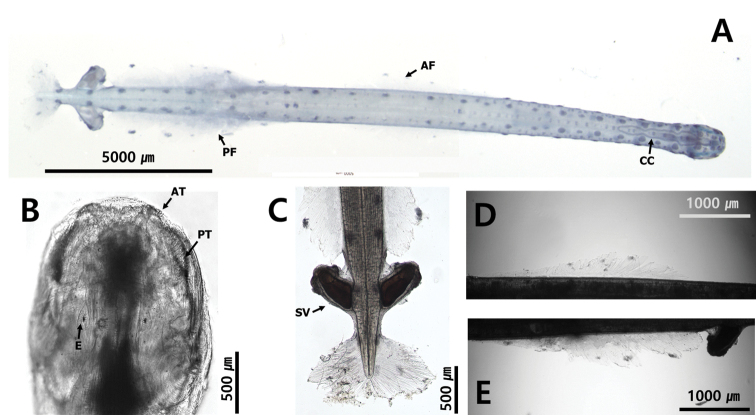
**A***Serratosagittapacifica* (dorsal view) **B** head **C** tail **D** anterior fin **E** posterior fin. Abbreviations: AF = anterior fin; AT = anterior teeth; CC = corona ciliata; COL = collarette; E = eye; PF = posterior fin; PT = posterior teeth; SV = seminal vesicle.

Figs 6F , 8C , 10C , 13A–E

Sagitta serratodentata pacifica : Tokioka, 1959: 72–80 p., fig. 10, table 10; Park et al. 1990: 52–54 p. figs 31, 32.
Sagitta pacifica : Alvariño, 1961: 71 p., fig. A, B, table 2; Alvariño 1967: 36–39 p., fig. 22A–D; Pierrot-Bults 1974: 221–222 p., fig. 6; Francisco 1977: 226–229 p., plate 1; Srinivasan 1979: 27–29 p., fig. 15A–G; Kim 1987: 18–20 p., plate 3.
Serratosagitta pacifica : Tokioka, 1965: 345–346 p.; Lutschinger 1993: 30–31 p., fig. 15 A–B.

##### Material examined.

Korea Strait (33°24.504'N, 127°54.600'E), 0–50 m depth, oblique towing with MOCNESS, May 2019, NIBRIV0000895311 (two specimens); Korea Strait (33°33.600'N, 127°34.002'E), 0–96 m depth, oblique towing with conical net, Feb 2020 (one specimen); northern East China Sea (32°33.000'N, 126°30.000 E), 0–100 m depth, oblique towing with conical net, Feb 2020, NIBRIV0000895310 (three specimens); northern East China Sea (32°00.000'N, 127°4.098'E), 0–120 m depth, oblique towing with conical net, Feb 2020, two specimens.

##### Description.

Total body length ranged within 11.8 and 13.7 mm. Tail 23.4–24.9% of body length. Hooks 6–7. Anterior 10–13 and posterior teeth 16–25. Rigid and opaque body (Fig. 13A). Small head (Fig. 13A, B). Grasping spines serrated on edge (Fig. 8C). Collarette absent (Fig. 10C, 13A). Rectangular eyes with “T” shaped eye pigments (Fig. 13B). Intestinal diverticula absent (Fig. 10C). Anterior fins spanned 21.9% of body length. Anterior fins completely rayed beginning between ventral ganglion and caudal septum. Starting point of anterior fins 34.6% and ending points of anterior fins 55.1% of body length, respectively (Fig. 13A, D). Posterior fins 26.2% of body length and 1.2 times longer than anterior fins. Starting points of posterior fins 63.7% and ending points of posterior fins 89.7% of body length, respectively. Posterior fins well-separated from anterior fins (Fig. 13A, E). Caudal fin triangular shaped (Fig. 13A, C). Seminal vesicles touched or closed to lateral fins and well-separated from tail fin (Fig. 13C) with elongated knob facing obliquely forward and teeth-like appendages forming 5–10 distal protrusions. Eggs reached anterior of anterior fins. Collarette beginning in front of eyes and extended over neck (Fig. 10C, 13A).

##### Distribution.

This species is found in the epipelagic (0–200 m depth) and mesopelagic zones (200–500 m depth) of the Pacific and Indian Oceans (Alvariño 1967; Pierrot-Bults and Nair 1991), the epipelagic zone of Red Sea, Californian waters (Pierrot-Bults 1976) and the Tosa Bay in Japan (Ohnishi et al. 2014). In this study, it was distributed in the epipelagic zone (0–50 m depth) of the Korea Strait and northern East China Sea (Fig. 1: stations KS06, KS07, nECS01 and nECS04).

##### Ecology.

This species mainly inhabits Indo-pacific warm-water masses (Bieri 1959). In the Pacific Ocean, it is a known indicator species of the Kuroshio Water Mass (Kim 1987). In this study, the temperature range of sampling locations was 16.37–20.57 °C and salinity ranged between 34.48–34.61 psu.

##### Remarks.

The seminal vesicles are used as an important morphological feature to identify the genus *Serratosagitta*. *S.serratodentata* has thick collarette tissue in front of the seminal vesicles and two projections at the anterior-lateral corner. The seminal vesicles touch the end of posterior fins (Alvariño 1961). The seminal vesicles of *S.pseudoserratodentata* have one projection at the front corner, with small teeth at the anterior end. The seminal vesicles are well-separated from the posterior fins and caudal fin (Alvariño 1961). In the *S.pacifica* (nine specimens), the number of protrusions vary between 5 and 10. The inner serrated row of the grasping spine and the “teeth cells” forming protrusions at the anterior margin of the seminal vesicles were consistent with previous records (Alvariño 1967; Pierrot-Bults 1976). We observed three specimens for CBE staining pattern: dorsomedian line 43 dots; dorsolateral line, 54–69 dots; lateral line, 24 dots; receptors on the lateral fin, 10 dots; anterolateral receptors on the caudal fin, 2 dots; posterior receptors on the caudal fin, 3–4 dots. The dorsomedian dots are patterned as small spots that cross the centre of the body and larger symmetrical spots on dorsolateral line dots.

#### ﻿Genus Mesosagitta

Taxon classificationAnimalia AphragmophoraSagittidae

Tokioka, 1965a

9015A71D-4377-5BA4-A9E4-672942DB948C

##### Diagnosis.

Flaccid and opaque body. Collarette absent. Intestinal diverticula present. Grasping spine gently curved and not serrated. Intestinal diverticula present. The anterior fins begin between ventral ganglion and caudal fin and are shorter than posterior fins.

#### ﻿ Mesosagitta minima

Taxon classificationAnimalia AphragmophoraSagittidae

(Grassi, 1881)

070AE91A-90A6-5703-BC9D-E837233A84BB

Spadella minima : Grassi, 1883: 15 p.
Sagitta minima : Aida, 1897: 15 p., fig. 5; Michael 1908: 74 p.; Michael 1919: 248–249 p.; Tokioka 1940: 5 p.; Thomson 1947: 19 p.; Prado 1961: 39–41 p.; Alvariño 1967: 59–61 p., figs 36, 37; Ducret 1974: 166 p., table.1; Srinivasan 1979: 24–26 p., fig. 13; Michel 1984: 25–26 p., fig. 34; McLelland 1989: 163 p., table.1, figs 8D, 12D.

##### Material examined.

Northern East China Sea (32°29.420'N, 127°29.654'E), 20–100 m depth, oblique towing with MOCNESS, Aug 2020 (one specimen).

##### Description.

Small head. Two pairs of lateral fins with rayless zone. Intestinal diverticula small. Seminal vesicles divided into a small anterior knob and elongated posterior part and well-separated from posterior fins, but touching caudal fin. Corona ciliata elongated oval-shaped beginning in neck.

##### Distribution.

This cosmopolitan species is found in the epipelagic zone (0–200 m depth) of the Pacific, Indian and Atlantic Oceans (Pierrot-Bults and Nair 1991) and the epipelagic zone (0–200 m depth) of the Japan coast (Sagami Bay and Suruga Bay) (Nagasawa and Marumo 1972). In this study, it was found in the epipelagic zone (20–100 m depth) of the northern East China Sea (Fig. 1, station nECS03).

##### Ecology.

*Mesosagittaminima* is abundant in mixed waters of the western North Atlantic Ocean (Pierce 1951). Mature specimens ranged within the size of 7–8 mm (Park 1970). The temperature range measured in the sampling stations was 18.49–28.84 °C and salinity range was 30.71–34.59 psu.

##### Remarks.

Only immature individuals could be collected in this study. We easily distinguished *M.minima* amongst the collected specimens by the relatively small head and unique body shape that thickens towards the tail. These Korean specimens were classified as immature because seminal vesicles and ovaries were absent and undeveloped short eggs (mentioned in description of Alvariño (1967) as a feature of the second stage of development) were observed in the body.

#### ﻿Genus Flaccisagitta

Taxon classificationAnimalia AphragmophoraSagittidae

Tokioka, 1965a

8120F184-607F-5C18-9FA7-B262532E1FE5

##### Diagnosis.

Transparent or translucent body. Collarette absent. Intestinal diverticula absent. The anterior fins begin at a far distance behind the end of the ventral ganglion. Seminal vesicles spherical shaped.

### ﻿Key to species of *Flaccisagitta*

**Table:** 

1	Large body (> 40 mm), small eggs reaching the neck	***F.hexaptera***
–	Small body (< 20 mm), large eggs reaching the anterior part of posterior fins	***F.enflata***

#### ﻿ Flaccisagitta enflata

Taxon classificationAnimalia AphragmophoraSagittidae

(Grassi, 1881)

51010C72-CEE2-5E0D-93BF-9A262FF18BA9

**Figure 14.**
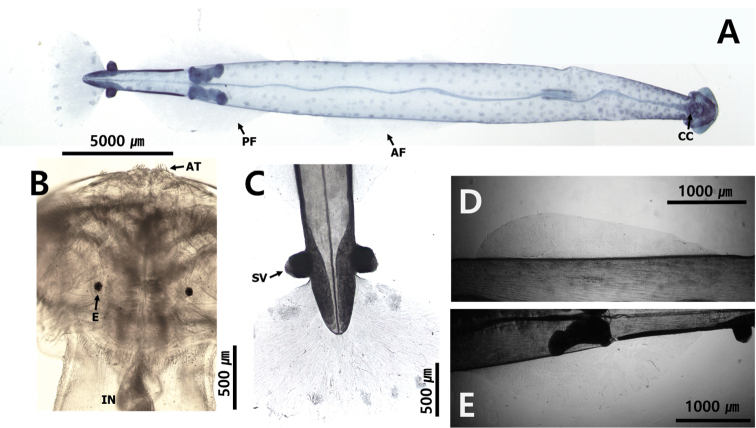
**A***Flaccisagittaenflata* (dorsal view) **B** head **C** tail **D** anterior fin **E** posterior fin. Abbreviations: AF = anterior fin; AT = anterior teeth; CC = corona ciliata; E = eye; IN = intestine; PF = posterior fin; SV = seminal vesicle.

Figs 3A , 6E , 10D and 14A–E

Spadella enflata : Grassi, 1883: 13 p., fig. 7.
Sagitta inflata : Ritter-Záhony, 1908: 13–15 p., fig. 4A–D; Srinivasan 1979: 18–19 p., fig. 9.
Sagitta enflata : Aida, 1897: 15–16 p., fig. 6; Fowler 1906: 69 p., figs 9–17; Ritter-Záhony 1909: 791–792 p.; Michael 1919: 242–244 p., fig. 28, table 1; Burfield and Harvey 1926: 95–96 p., fig. 5; Pierce 1951: 221–222 p., fig. 4, table 12; Alvariño 1967: 29–34 p., fig. 17A–G; Michel 1984: 18–19 p., figs 2, 20.
Sagitta enflata f. gardineri : Tokioka, 1959: 91–92 p., table 19
Flaccisagitta enflata : McLelland, 1989: 159 p., figs 7A and 12B

##### Material examined.

Korea Strait (32°59.175'N, 124°29.595'E), 20–25 m depth, oblique towing with MOCNESS, Nov 2019, NIBRIV0000895309 (three specimens); northern East China Sea (32°0.000'N, 127°4.098'E), 0–110 m depth, oblique towing with conical net, Feb 2020, NIBRIV0000895308 (four specimens); northern East China Sea (32°30.000'N, 126°30.000'E), 0–100 m depth, oblique towing with conical net, Feb 2020 (one specimen).

##### Description.

Total body length ranged between 12.7 and 15.4 mm. Tail 14.1–17.6% of body length. Hooks 8–10. Anterior teeth 6–10 and posterior teeth 10–17, respectively. Transparent body, inflated towards middle (Fig. 14). Triangular-shaped head (Fig. 14A). Collarette absent (Figs 3A, 10D). Blunt teeth (Fig. 14B). Round eyes with star-shaped eye pigments (Fig. 14B). Intestinal diverticula absent (Fig. 10D). Anterior fins 17.0% of body length. Anterior fins began at middle of body at far distance back of ventral ganglion and partially rayed. Starting points of anterior fins 43.7% and ending points of anterior fins 64.4% of body length, respectively (Fig. 14A, D). Posterior fins 20.5% of body length and 1.2 times longer than anterior fins. Starting points of posterior fins 71.9% and end points of posterior fins 92.5% of body length, respectively. Posterior fins well-separated from anterior fins (Fig. 14A, E). Caudal fin roundish, fan-shaped and fully rayed (Fig. 14C). Seminal vesicles touching caudal fin, but separated from posterior fins, spherical in shape with rupture in middle in mature specimen (Fig. 14C). Corona ciliata beginning in front of eyes and reaching neck (Figs 10D, 14A, 14B).

##### Distribution.

This cosmopolitan species is found in the epipelagic (0–200 m depth) and mesopelagic zones (200–500 m depth) of the Pacific, Indian and Atlantic Oceans (Pierrot-Bults and Nair 1991), the coastal area of Japan (Tosa Bay; Ohnishi et al. 2014) and the epipelagic zone (0–150 m depth) of the Korea Strait (Park 1970). In this study, it was found in the epipelagic zone (0–110 m depth) of the northern East China Sea (Fig. 1: stations KS08, nECS01 and nECS04).

##### Ecology.

This is used as an indicator species of warm currents in water surrounding Korea (Park 1970). The temperature range in the sampling stations was 16.52–28.80 °C and the salinity range was 28.96–33.22 psu.

##### Remarks.

The transparent and flaccid body, star-shaped eye pigments and seminal vesicle morphology were consistent with those recorded in previous studies by Alvariño (1967) and Nagasawa (1976). Two types of Korean *Flaccisagittaenflata* have been reported, a small type: 10–20 mm long and a large type: 20–28 mm long (Park 1970). In this study, only the small type (< 20 mm) of *F.enflata* was collected. We observed seven specimens for CBE staining pattern: dorsomedian line, 12 dots; dorsolateral line, > 150 dots; ambiguous lateral line, receptors on the lateral fin, 2 dots (easily damaged); anterolateral receptors on the caudal fin, 4 dots; posterior receptors on the caudal fin, 7 dots. The pattern of dorsomedian dots lined up behind the ventral ganglion and the pattern of dorsolateral dots intensively scattered ahead of ventral ganglion.

#### ﻿ Flaccisagitta hexaptera

Taxon classificationAnimalia AphragmophoraSagittidae

(d’Orbigny, 1836)

BE8140D1-5EDD-51BA-AD65-4C0A9365252E

Fig. 15A–E

Sagitta hexaptera : Conant, 1896: 213 p.; Aida 1897: 14 p., fig. 3; Fowler 1906: 70 p., figs 30–33; Ritter-Záhony 1908: 9–10 p., figs 3, 3A, 3B; Ritter-Záhony 1909: 789–790 p.; Ritter-Záhony 1911: 2–3 p.; Burfield and Harvey 1926: 95–96 p., figs 6–9; Tokioka 1959: 382–383 p., fig. 21; Alvariño 1967: 27–29 p., fig. 16A–I; Srinivasan 1979: 14–16 p., fig. 7A–G; Michel 1984: 21p., fig. 25.
Flaccisagitta hexaptera : McLelland, 1989: 159–160 p., figs 7B, C and 12A.

##### Material examined.

Korea Strait (33°24.504'N, 127°54.600'E), 0–50 m depth, oblique towing with MOCNESS, Nov 2019, NIBRIV0000895298 (one specimen).

##### Description.

**Figure 15.**
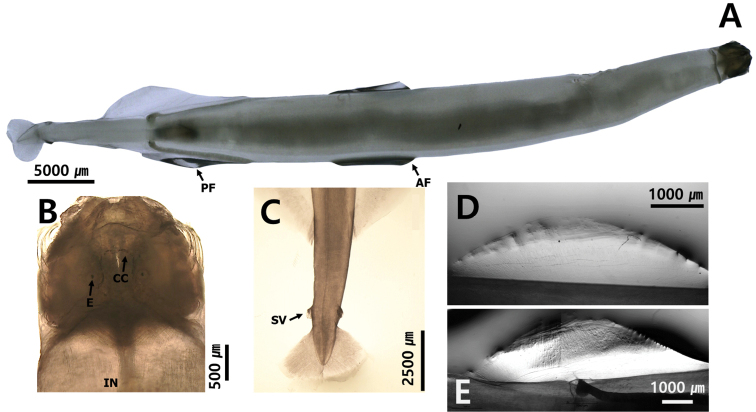
**A***Flaccisagittahexaptera* (dorsal view) **B** head **C** tail **D** anterior fin **E** posterior fin. Abbreviations: AF = anterior fin; CC = corona ciliata; E = eye; IN = intestine; PF = posterior fin; SV = seminal vesicle.

Total body length ranged between 15 and 49 mm. Tail 19–24% of body length. Hooks 4–11. Anterior teeth 2–4 and posterior teeth 2–9, respectively. Large and translucent body (Fig. 15). Intestinal diverticula absent (Fig. 15B). Collarette absent (Fig. 15A). Eyes “D” shaped with “T” shaped eye pigments (Fig. 15B). Anterior fins short, beginning at middle of body between ventral ganglion and caudal septum, round-shaped and partially rayed (Fig. 15A, D). Posterior fins well-separated from anterior fins and partially rayed (Fig. 15A, E). Caudal fin roundish triangular-shaped and completely rayed (Fig. 15A, C). Seminal vesicles spherical with anterolateral edge opening (Fig. 15C). Seminal vesicle touching or close to tail fin and well-separated from lateral fins (Fig. 15C). Eggs reaching forward end of anterior fins. Corona ciliata beginning in front of eyes and reaching neck (Fig. 15B).

##### Distribution.

This cosmopolitan species is found in the epipelagic (0–200 m depth) and mesopelagic zones (200–500 m depth) of the Pacific, Indian and Atlantic Oceans (Pierrot-Bults and Nair 1991), in the Indian coast (George 1952), the Tosa Bay in Japan (Ohnishi et al. 2014), the Korea Strait and south of East Sea (Park 1970). In this study, it was found in the epipelagic zone (0–50 m depth) of the Korea Strait (Fig. 1: station KS07).

##### Ecology.

This species is considered as an indicator species of the Kuroshio warm current and fully-grown adults inhabit depths of < 200 m (Park 1970). In this study, individuals under Stage 3 were mainly found between the Jeju Straits (0–100 m depth). The temperature range in the sampling stations was 16.52–20.57 °C and salinity range was 34.48–34.61 psu.

##### Remarks.

Though mainly immature individuals were reported in previous studies from Korea, in this study, adults longer than 40 mm were collected for the first time. The Korean species was consistent with those found in previous studies by Alvariño (1967) and Michel (1984) in terms of body size, length and shape of the egg, presence of small and round anterior fins in the middle of the body and the absence of bridge connecting the anterior and posterior fins. As the adult *Flaccisagittahexaptera* is large (> 20 mm), it can be difficult to distinguish the adult *Flaccisagittaenflata* from the immature *F.hexaptera*. However, it is possible when noting that the eggs of *F.hexaptera* are long and thin, whereas those of *F.enflata* are large and short. Another feature relevant for the diagnosis of *F.hexaptera* is the number of conspicuous anterior teeth which never exceed four, while it is eight in *F.enflata*. No specific pattern was observed through CBE staining on the body surface.

#### ﻿Genus Pseudosagitta

Taxon classificationAnimalia AphragmophoraSagittidae

Germain & Joubin, 1912

E6797681-43AD-528A-905F-ABF8CA35E246

##### Diagnosis.

Flaccid and transparent body (but more opaque than *Flaccisagitta*). Collarette absent. Intestinal diverticula absent. Two pairs of lateral fins partially rayed and connected with a tegumentary bridge. Anterior fins beginning at the rear end of ventral ganglion and longer than posterior fins.

#### ﻿ Pseudosagitta lyra

Taxon classificationAnimalia AphragmophoraSagittidae

(Krohn, 1853)

C3CDEEDD-3159-5B85-BD01-82503ED2B93C

Figs 6C , 16A–E

Sagitta lyra : Aida, 1897: 15 p., fig. 4; Fowler 1906: 33 p.; Ritter-Záhony 1908: 10–13 p., fig. 1A–E; Burfield and Harvey 1926: 98 p., figs 18–24; Thomson 1947: 10–11 p.; Alvariño 1967: 23–26 p., fig. 14A–O; Lea 1955: 28–30 p., plate 3; Srinivasan 1979: 20–21 p., fig. 10A–F; Michel 1984: 22–23 p., fig. 27.
Flaccisagitta lyra : McLelland, 1989: 162 p., figs 7D, 7E and 12C.

##### Material examined.

Korea Strait (33°48.924'N, 126°48.666'E), 40–70 m depth, oblique towing with MOCNESS, May 2019, NIBRIV0000895307 (one specimen).

##### Description.

**Figure 16.**
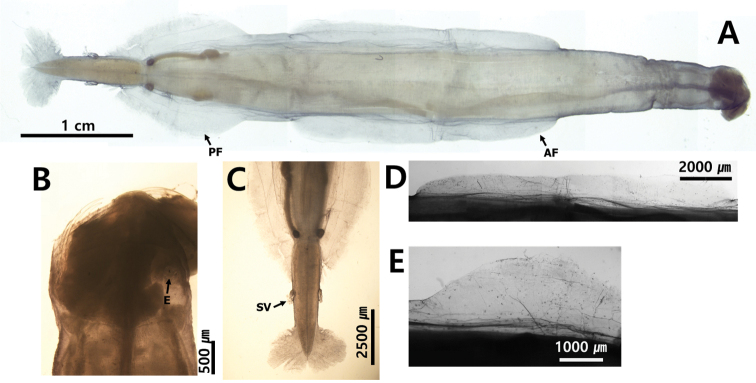
**A***Pseudosagittalyra* (dorsal view) **B** head **C** tail **D** anterior fin **E** posterior fin. Abbreviations: AF = anterior fin; E = eye; PF = posterior fin; SV = seminal vesicle.

Total body length ranged between 16.0 and 60.0 mm. Tail 14–20% of body length. Hooks 8–9. Anterior teeth 4–9 and posterior 8–10, respectively. Large, flaccid and opaque body (Fig. 16). Intestinal diverticula absent (Fig. 16B). Collarette absent (Fig. 16A and B). Eyes square shaped with “H” shaped eye pigments (Fig. 16B). Anterior fins beginning at ventral ganglion, anterior of anterior fins with ray less zone and angular shape, conspicuously longer than posterior fins (Fig. 16A, D). Posterior fins with rayless zone connected with anterior fins by tegumentary bridge (Fig. 16A, E). Caudal fin roundish and completely rayed (Fig. 16C). Eggs reaching middle of posterior fins (Fig. 16A). Corona ciliata not clear (Fig. 16B). Seminal vesicles spherical and opening in middle of edge (Fig. 16C). Seminal vesicles touching neither of posterior or caudal fins, located closer to posterior fins (Fig. 16C).

##### Distribution.

This species is found in the mesopelagic (500–1,000 m depth) and bathypelagic zones (1,000–2,000 m depth) of the Pacific, Indian and Atlantic Oceans (Pierrot-Bults and Nair 1991) and the Tosa Bay in Japan (Ohnishi et al. 2014). In this study, it was distributed in the epipelagic zone (40–70 m depth) of the Korea Strait (Fig. 1, station KS06).

##### Ecology.

This species has a high prevalence in warm waters (Park 1970). In this study, specimens collected around Jeju Island were mainly distributed at water depths > 50 m. The temperature range in the sampling stations was 16.47–21.34 °C and the salinity range was 34.17–34.52 psu.

##### Remarks.

In Korean specimens, the position and length of the fins, distance between the anterior and posterior fins and shape of the seminal vesicles were morphologically consistent with the previous records of *Pseudosagittalyra* (Alvariño 1967; Michel 1984; Lutschinger 1993). As one of the larger species of arrow worm, *P.lyra* reaches a maximum size of 42 mm (Michel 1984; Lutschinger 1993). However, the largest of the Korean specimens collected in this study was 60 mm in length. *Pseudosagittascrippsae* can be easily confused with *P.lyra*, with similar size and position and shape of the fins and seminal vesicles. However, *P.scrippsae* can be differentiated by the presence of a distinct collarette around the neck (Chihara and Murano 1997). No specific pattern was observed through CBE staining on the body surface.

#### ﻿Genus Parasagitta

Taxon classificationAnimalia AphragmophoraSagittidae

Tokioka, 1965a

FD4C6872-1955-52A8-919F-3D2079FE1C19

##### Diagnosis.

Slender and either opaque or translucent body. Collarette absent or small (almost absent). Intestinal diverticula present or absent. Grasping spines not serrated. Two rows of teeth. Two pairs of lateral fins completely rayed.

#### ﻿ Parasagitta elegans

Taxon classificationAnimalia AphragmophoraSagittidae

(Verrill, 1873)

E3E44616-BFA9-5AF6-9B3B-402BF57B6763

Fig. 17A–E

Sagitta elegans : Verrill, 1873: 332–333 p.; Conant 1896: 211–212 p.; Fowler 1906: 31–32 p.; Lea 1955: 22–28 p., plate 3.

##### Material examined.

East Sea (37°33.198'N, 131°14.598'E), 0–100 m depth, oblique towing with conical net, Feb 2020, NIBRIV0000895306 (five specimens).

##### Description.

**Figure 17.**
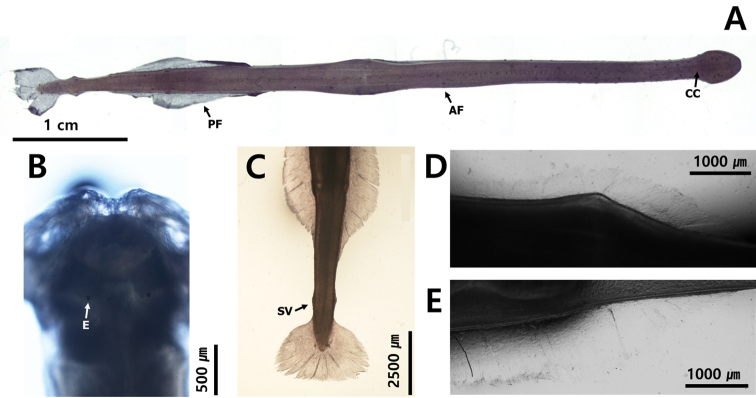
**A***Parasagittaelegans* (dorsal view) **B** head **C** tail **D** anterior fin **E** posterior fin. Abbreviations: AF = anterior fin; E = eye; PF = posterior fin; SV = seminal vesicle.

Total body length ranged between 32.5 and 37.0 mm. Tail 14.7–20.6% of body. Hooks 11–12. Anterior teeth 9–10 and posterior teeth 22–29, respectively. Rigid and opaque body (Fig. 17). Collarette absent (Fig. 17A). Intestinal diverticula present, but not obvious. Anterior fins 18.8% of body length. Anterior fins beginning at middle of ventral ganglion and partially rayed. Starting points of anterior fins 38.5% and ending points of anterior fins 57.5% of body length, respectively (Fig. 17D). Posterior fins 22.3% of body length and 1.2 times longer than anterior fins. Starting points of posterior fins 67.7% and ending points of posterior fins 88.7% of body length, respectively. Posterior fins well-separated from anterior fins (Fig. 17E). Seminal vesicles elongated (Fig. 17C). Caudal fin roundish triangular-shaped (Fig. 17C). Square eyes “+” shaped eye pigments (Fig. 17B). Corona ciliata beginning in front of eyes and expanding to anterior trunk (Fig. 17B).

##### Distribution.

This species is found in the Epipelagic (0–200 m depth), mesopelagic (200–500 m depth) and bathypelagic zones (1000–2000 m depth) of the Pacific, Indian and Atlantic Oceans (Terazaki 1998; Choe and Deibel 2000) and the epipelagic and mesopelagic zones of the East Sea (Park 1970). In this study, it was found in the epipelagic zone (0–100 m depth) of the East Sea.

##### Ecology.

A cold-water species, *P.elegans* is mainly found in the northern part of the Pacific Ocean (Bieri 1959). The spawning season is winter and fully mature individuals are ≥ 30 mm in length (Park 1970). The temperature range in the sampling stations of this study was 8.20–11.97 °C and the salinity range was 34.11–34.20 psu.

##### Remarks.

The absence of a rayless zone in the anterior and posterior fins and a collarette in anterior body and the presence of small seminal vesicles extending along the body in the Korean specimens of *Parasagittaelegans* were consistent with previous records (Chihara and Murano 1997). Adult specimens (> 40 mm) collected in this study had small intestinal diverticula. CBE staining showed a spot pattern dividing the centre from the head to the tail septum. We observed one specimen for CBE staining pattern: dorsomedian line small, 60 dots; dorsolateral line not observed due to damage; lateral line, 42 dots; receptors on the lateral fin not found; anterolateral receptors on the caudal fin, 2 dots; posterior receptors on the caudal fin not found.

#### ﻿Genus Ferosagitta

Taxon classificationAnimalia AphragmophoraSagittidae

Kassatkina, 1971

1DBDF129-C0B9-5FE1-8238-D1593F46324B

##### Diagnosis.

Rigid and opaque body. Collarette present. Intestinal diverticula present. Grasping spines not serrated. Two pairs of lateral fins completely or partially rayed.

### ﻿Key to species of *Ferosagitta*

**Table:** 

1	Seminal vesicles oval shaped, posterior fins with small rayless zone	***F.ferox***
–	Seminal vesicles pear shaped, anterior and posterior fins fully rayed	***F.robusta***

#### ﻿ Ferosagitta ferox

Taxon classificationAnimalia AphragmophoraSagittidae

(Doncaster, 1902)

F832560A-2D50-53EB-BD83-2E8D8248DBE8

Sagitta ferox : Doncaster, 1902: 212 p.; Fowler 1906: 10–11 p.; Michael 1919: 259–262 p., tables 14–15; Tokioka 1959: 353–358 p., tables 1–4, figs 1–3; Alvariño 1962: 189–190 p., tables 1–5, figs 6–10; Alvariño 1967: 66–67 p., table 11, figs 40–41; Srinivasan 1979: 13–14 p., fig. 6.

##### Material examined.

Korea Strait (33°24.504'N, 127°54.600'E), 0–50 m depth, oblique towing with MOCNESS, May 2019 (one specimen).

##### Description.

Broad head. Rigid and opaque body. Collarette present. Intestinal diverticula present. Grasping spines gently curved. Two rows of stout teeth arranged in comb shape. Two pairs of lateral fins partially rayed, anterior fins beginning at middle of ventral ganglion. Seminal vesicles oval-shaped with an anterior protruding part touching both lateral and caudal fins.

##### Distribution.

This species is found in the epipelagic zone (0–200 m depth) of the Pacific and Indian Oceans (Pierrot-Bults and Nair 1991), the coast of Japan (Tosa Bay) (Ohnishi et al. 2014) and the epipelagic zone (0–100 m depth) of the Korea Strait (Park 1970). In this study, it was found in the epipelagic zone (0–100 m depth) in Korea Strait (Fig. 1, station KS07).

##### Ecology.

This species inhabits the surface layer of the warm water and is mainly dominant in the Kuroshio Current of the Japanese waters (Chihara and Murano 1997). In this study, the temperature range in the sampling locations was 25.87–28.70 °C and the salinity range was 32.72–33.11 psu.

##### Remarks.

Only immature individual was collected in this study. We easily distinguished *F.ferox* amongst the collected specimens by the presence of distinct head as wide as the trunk and the presence of elongated ovoid seminal vesicles on the body. The Korean specimen was classified as immature because the boundary of the seminal vesicles was not obvious and the inside was mostly empty. This characteristic is consistent with Alvariño’s (1967) description regarding the immaturity of this species.

#### ﻿ Ferosagitta robusta

Taxon classificationAnimalia AphragmophoraSagittidae

(Doncaster, 1902)

D6DEC187-CB62-5498-9887-280D24B788AC

**Figure 18.**
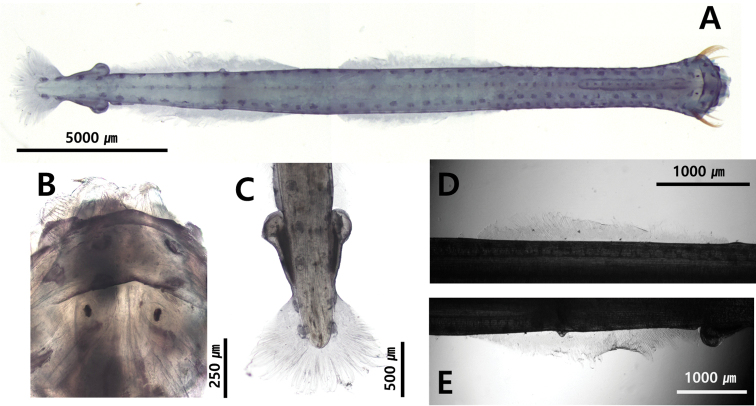
**A***Ferosagittarobusta* (dorsal view) **B** head **C** tail **D** anterior fin **E** posterior fin. Abbreviations: AF = anterior fin; AT = anterior teeth; CC = corona ciliata; E = eye; PF = posterior fin; PT = posterior teeth; SV = seminal vesicle.

Figs 3A , 6B , 8B , 9A , 18A–E

Sagitta robusta : Fowler, 1906: 19–20 p., figs 59–64; Ritter-Záhony 1908: 792 p.; Burfield and Harvey 1926: 100–101 p., figs 33–37; Thomson 1947: 13–15 p.; Alvariño 1962: 187–198 p., figs 1–5; Alvariño 1967: 66–71 p., figs 42A–D; Srinivasan 1979: 32–34 p., fig. 18A–G.

##### Material examined.

Yellow Sea (34°5.502'N, 124°36.000'E), 0–75 m depth, oblique towing with conical net, Aug 2019, NIBRIV0000895305, (one specimen).

##### Description.

Total body length ranged between 10.0 and 11.5 mm. Tail 27.8–35.0% of body length. Hooks 6–8. Anterior teeth 9–10 and posterior teeth 12–20, respectively. Rigid and opaque body (Fig. 18). Head wide as neck, roughly round or square shaped (Fig. 18A). Grasping spines gently curved and not serrated edge of hooks (Figs 8B, 9A). Teeth short and firm (Fig. 9A). “B” shaped eyes had “T” shaped eye pigments (Fig. 18B). Collarette on neck (Figs 9A, 18A). Anterior fins 22.4% of body length. Anterior fins beginning just at posterior of ventral ganglion. Starting points of anterior fins 33.1% and ending points of anterior fins 56.5% of body length, respectively (Fig. 18A, D). Posterior fins 25.8% of body length and 1.2 times longer than anterior fins. Starting points of posterior fins 62.6% and ending points of posterior fins 88.4% of body length, respectively. Posterior fins separated from anterior fins and partially rayed. Caudal fin roughly round-triangle shaped, completely rayed (Fig. 18C). Seminal vesicles pear-shaped with elongated posterior trunk and anterior roundish knob and touching both posterior and caudal fins. (Fig. 18A, C). Conspicuous intestinal diverticula present (Fig. 9A). Eggs reached anterior of ventral ganglion. Corona ciliata beginning behind eyes and expanding to anterior of trunk (Fig. 18A).

##### Distribution.

This species is found in the epipelagic zone (0–200 m depth) of Pacific and Indian Oceans (Pierrot-Bults and Nair 1991), the west coast of Florida (Pierce 1951), Indian coast (George 1952) and the Tosa Bay in Japan (Ohnishi et al. 2014). In this study, specimens were found in the epipelagic zone (0–75 m depth) of the Yellow Sea (Fig. 1: station YS03). *Ferosagittarobusta* is a typical Indo-pacific warm-water species (George 1952). The horizontal distribution range is wider than the vertical range and many individuals are mainly found in the surface layer (Park 1970). In this study, this species rarely appeared under low temperature conditions and their presence was predominant in the sea area affected by warm currents. The temperature range in the sampling stations was within 25.87–28.70 °C and salinity range was 32.72–33.11 psu.

##### Remarks.

Characteristics of the species in Korean waters, such as the conspicuous intestinal diverticula, head and body width and the seminal vesicles shape, are consistent with previous records (Alvariño 1967; Chihara and Murano 1997). The main difference between this species and another congeneric species of Korea, *F.ferox*, is the shape of the seminal vesicles. Seminal vesicles of *F.robusta* are very conspicuous, touching both posterior end of posterior fins, while that in *F.ferox* are not so conspicuous and are close to both posterior fins and caudal fin (Alvariño 1962). We observed one specimen for CBE staining pattern: dorsomedian line, 49 dots; dorsolateral line, 38 dots; lateral line, 30 dots; receptors on the lateral fin, 6 dots; anterolateral receptors on the caudal fin, 4 dots; posterior receptors on the caudal fin not observed due to damage.

#### ﻿Genus Aidanosagitta

Taxon classificationAnimalia AphragmophoraSagittidae

Tokioka & Pathansali, 1963

6CA6EFAD-D214-5DB6-B5E4-44BC5FEDB715

##### Diagnosis.

Rigid and opaque body. Intestinal diverticula present. Collarette present or absent. Grasping spine gently curved and not serrated. Two rows of stout teeth arranged in a comb shape. Two pairs of lateral fins completely rayed.

### ﻿Key to species of *Aidanosagitta*

**Table:** 

1	Seminal vesicles large and oval shaped, well-separated from caudal fins but touching posterior fins	***A.neglecta***
–	Seminal vesicles small and elongated	**2**
2	Seminal vesicles touching both posterior fins and caudal fin and opening at the anterolateral edge. Collarette covered on ventral ganglion (N type) from head to body (C type) or on the partial body (I type)	***A.crassa***
–	Seminal vesicles well-separated from caudal fins, but touching the posterior fins. Thick collarette covered head to tail. Small sized body (< 10 mm)	***A.regularis***

#### ﻿ Aidanosagitta neglecta

Taxon classificationAnimalia AphragmophoraSagittidae

(Aida, 1897)

7DF80D6C-50EB-51AA-9F22-A4E664DD3191

Sagitta neglecta : Aida, 1897: 16–17 p., fig. 7; Fowler 1906: 15–17 p., fig. 8; Michael 1919: 258 p., table 13, fig. 9; Burfield and Harvey 1926: 99 p, fig. 27; Thomson 1947: 17–18 p.; Tokioka 1959: 373–375 p., table 12, figs 103, 104; Sund 1961: 110 p., table 1; Alvariño 1967: 74 p., table 12, figs 46, 47; Srinivasan 1979: 26–27 p., fig. 14; Nair et al. 2008: 210 p., table 2.

##### Material examined.

Korea Strait (34°41.577'N, 127°50.460'E), 0–20 m depth, oblique towing with conical net, Feb 2021 (one specimen).

##### Description.

Rigid and opaque body. Narrow collarette and extending to half distance from neck to ventral ganglion. Intestinal diverticula present. Grasping spine gently curved. Two rows of stout teeth arranged in comb shape. Two pairs of lateral fins completely rayed, anterior fins beginning at end of ventral ganglion. Seminal vesicles oval-shaped with opening at anterolateral edge, position of seminal vesicles well separated from caudal fin, but touching posterior fins.

##### Distribution.

This species is found in the epipelagic zone (0–200 m depth) of the Pacific and Indian Oceans (Pierrot-Bults and Nair 1991) and the coastal waters of the Philippines, India and Hong Kong (George 1952; Tse et al. 2007; Noblezada and Campos 2008; Lie et al. 2012).

##### Ecology.

A mature specimen has been reported to be 8 mm is size (Alvariño 1967). This species shows a diurnal vertical migration in the summer off the Hong Kong coast (Lie et al. 2012). The temperature range in the sampling stations of this study was 16.40–16.41 °C and the salinity was 34.58 psu.

##### Remarks.

Only one immature individual was collected in this study. *Aidanosagittaneglecta* is similar to *A.regularis* in morphological characteristics including collarette. However, the former had much larger seminal vesicles, thus they can be easily distinguished from each other. *Aidanosagittaneglecta* collected from Korean waters was smaller than the previously reported adult specimens, but seminal vesicles were obviously swollen in an oval shape. Despite the presence of swollen seminal vesicles, the Korean specimen was considered as immature because its size was smaller than the known record of the adult (Alvariño 1967).

#### ﻿ Aidanosagitta crassa

Taxon classificationAnimalia AphragmophoraSagittidae

(Tokioka, 1939)

EAF6ACF9-7B10-5A92-BFE8-E99FD96875EC

**Figure 19.**
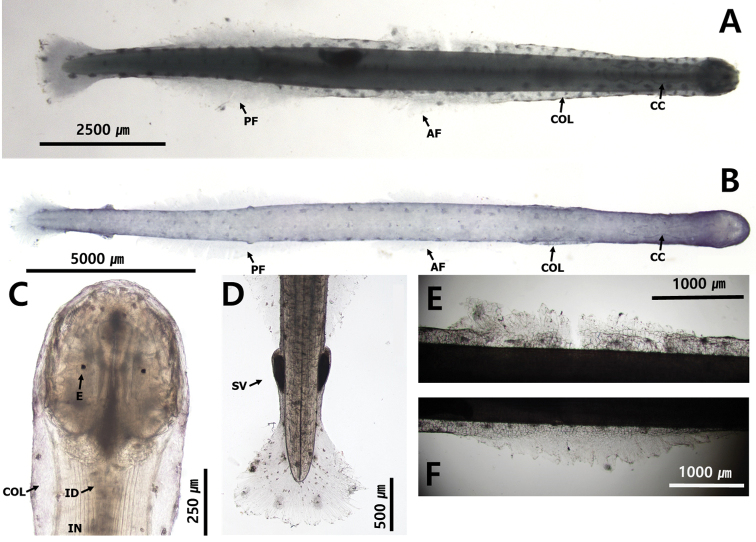
**A***Aidanosagittacrassa* Type C (dorsal view) **B***Aidanosagittacrassa* Type N (dorsal view) **C** head and neck **D** tail **E** anterior fin **F** posterior fin. Abbreviations: AF = anterior fin; CC = corona ciliata; COL = collarette; E = eye; IN = intestine; ID = intestine diverticula; PF = posterior fin; SV = seminal vesicle.

Figs 3C , 5B , 19A–F

Aidanosagitta crassa f. naikaiensis : Tokioka, 1959: 376–377 p., fig. 16, table 15.
Sagitta crassa : Tokioka, 1939: 349–352 p., figs 1–8

##### Material examined.

**Type C (collarette type)**: East Sea (37°33.198'N, 131°14.598'E), 0–100 m depth, oblique towing with conical net, Feb 2020, NIBRIV0000895304 (one specimen); Yellow Sea (35°22.550'N, 126°5.366'E), 0–16.5 m depth, oblique towing with conical net, Jul 2020, five specimens; Korea Strait (34°13.698'N, 127°35.400'E), 0–28 m depth, oblique towing with conical net, Feb 2020 (one specimen). **Type N (naked type)**: Yellow Sea (35°17.316'N, 126°10.483'E), 0–6.4 m depth, oblique towing with conical net, Jul 2020, NIBRIV0000895303 (three specimens).

##### Description.

**Type C**: total body length ranged within 9.9 and 11.2 mm. Tail 26.6–29.8% of body length. Rigid and opaque body (Fig. 19). Head small. Collarette beginning at neck and reaching middle of body (Fig. 19A). Round eyes star-shaped eye pigments. Corona ciliata beginning from neck, elongated to anterior of ventral ganglion (Fig. 19A, B). Intestinal diverticula present (Fig. 19C). Two pairs of lateral fins completely rayed (Fig. 19E, F). Anterior fins 18.1% of body length. Anterior fins beginning at posterior of ventral ganglion. Starting points of anterior fins 29.6% and ending points of anterior fins 46.6% of body length, respectively (Fig. 19D). Posterior fins 23.0% of body length and 1.3 times longer than anterior fins. Starting of posterior fins 62.0% and ending points of posterior fins 86.9% of body length, respectively. Posterior fins well separated from anterior fins beginning at middle of body (Fig. 19A, D). Seminal vesicles longitudinally elongated (Fig. 19D). Eggs reaching posterior of ventral ganglion.

**Type N**: total body length ranged between 8.1 and8.2 mm. Tail 27.0–30.0% of body length (Fig. 19B). Collarette beginning at anterior of ventral ganglion and reaching posterior of ventral ganglion (Fig. 19B). Anterior fins 19.1% of body length. Anterior fins beginning at posterior of ventral ganglion. Starting points of anterior fins 34.0% and end points of anterior fins 54.6% of body length, respectively (Fig. 19B). Posterior fins 24.6% of body length and 1.3 times longer than anterior fins. Starting points of posterior head 60.9% and ending points of posterior fins 88.2% of body length, respectively. Posterior fins well-separated from anterior fins beginning at middle of body (Fig. 19A, B).

##### Distribution.

This species is found in the neritic water of the Pacific Ocean (Pierrot-Bults and Nair 1991), the neritic coastal water of Hong Kong (Tse et al. 2007) and the Tosa Bay of Japan (Ohnishi et al. 2014). In this study, it was found in the epipelagic zone (0–100 m depth) of the East Sea, Korea Strait and Yellow Sea (Fig. 1, stations ES01, YS01, YS02 and KS01).

##### Ecology.

*Aidanosagittacrassa* appears in high abundance throughout the year in the relatively low saline waters of the Yellow Sea and coast of Jeju (Park 1970). This species rarely appears in the summer warm waters of southern Korea. The body length varies according to the season and it has been reported that they are large (type C) in winter and small in summer (type N) (Park 1970). Specimens of type C die after spawning and those of type N dominate the new generation (Park 1970). We obtained specimens from the East Sea in winter and the Yellow Sea in summer.

##### Remarks.

Previous researchers classified *Aidanosagittacrassa* into three types according to the distribution of the collarette: C type, covers from the neck to the body; N type, covers only the ventral ganglion; and I type, covers the ventral ganglion and partially covers the body (Park 1970). In this study, specimens of types C and N were collected and the Korean type C from the Yellow Sea had the same morphological characteristics of the collarette as the original description of this species reported by Tokioka (1939). Similarly, type N, which appeared together with type C at other stations of the Yellow Sea, was consistent with the morphological characteristics of the collarette of *A.crassa* and *Aidanosagittacrassaf.naikaiensis* (Tokioka 1939). It has been reported that the three types of *A.crassa* appear at different periods depending on environmental factors (water temperature and salinity) of the specific sea area; however, in this study, both types appeared simultaneously in the Yellow Sea. A more detailed ecological investigation of the impact of environment factors on the succession of the three types of *A.crassa* is necessary. We observed four specimens for type C of *A.crassa*CBE staining pattern: dorsomedian line, 14 dots; dorsolateral line, > 100 dots; lateral line, 8 dots; receptors on the lateral fin, 8 dots; anterolateral receptors on the caudal fin, 4 dots; posterior receptors on the caudal fin, 6 dots. The pattern of dorsomedian dots was small spots that crossed the centre of the body and larger symmetrical spots on dorsolateral line dots. In addition, we observed three specimens for type N of *A.crassa*CBE staining pattern: dorsomedian line, 35 dots; dorsolateral line, 34 dots; lateral line, 12 dots; receptors on the lateral fin, not observed; anterolateral receptors on the caudal fin, 2 dots; posterior receptors on the caudal fin, 4 dots.

#### ﻿ Aidanosagitta regularis

Taxon classificationAnimalia AphragmophoraSagittidae

(Aida, 1897)

ACA7B54C-85E1-55CD-AA86-38C29B02F1F8

**Figure 20.**
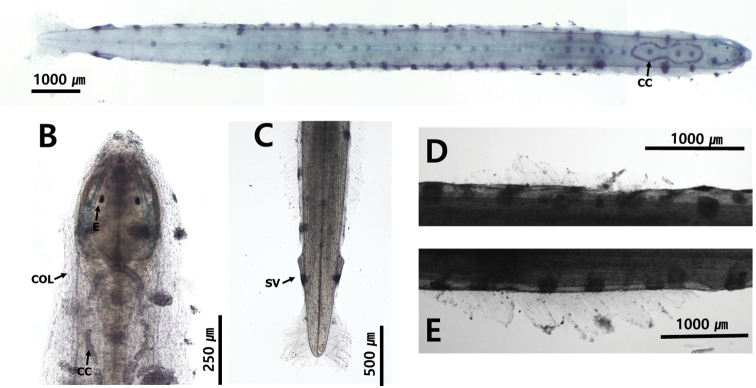
**A***Aidanosagittaregularis* (dorsal view) **B** head **C** tail **D** anterior fin **E** posterior fin. Abbreviations: CC = corona ciliata; COL = collarette; E = eye; SV = seminal vesicle.

Figs 6A , 7A , 10A , 20A–E

Sagitta regularis : Aida, 1897: 17–18 p., fig. 8; Doncaster 1902: 211 p., fig. 7; Burfield and Harvey 1926: 100 p., figs 31–32; Thomson 1947: 18–19 p.; Alvariño 1967: 72–75 p., fig. 48A–D; Srinivasan 1979: 31–32 p., fig. 17A–F; Nair et al. 2008: 110 p., table 2.

##### Material examined.

Korea Strait (33°33.600'N, 127°34.002'E), 0–96 m depth, oblique towing with conical net, Feb 2020, NIBRIV0000895302 (one specimen); northern East China Sea (32°33.000'N, 126°30.000'E), 0–100 m depth, oblique towing with conical net, Feb 2020, NIBRIV0000895301 (one specimen); northern East China Sea (32°30.000'N, 127°5.100'E), 0–120 m depth, oblique towing with conical net, Feb 2020 (one specimen).

##### Description.

Total body length ranged within 6.1 and 6.7 mm. Tail 30.5–31.7% of body length. Hooks 6. Anterior teeth 3–4 and posterior teeth 5–6, respectively. Rigid and opaque body (Fig. 20). Head small, triangular shaped (Fig. 20B). Round eyes with “B”-shaped eye pigments (Fig. 20B). Collarette expanding to seminal vesicles (Fig. 20A). Corona ciliata beginning from neck to anterior of ventral ganglion (Fig. 10A). Anterior fins 12.3% of body length. Anterior fins beginning at posterior of ventral ganglion. Starting points of anterior fins 37.3% and ending points of anterior fins 50.9% of body length, respectively (Fig. 20D). Posterior fins 22.0% of body length and 1.8 times longer than anterior fins. Starting points of posterior fins 62.9% and ending points of posterior fins 84.7% of body length, respectively. Posterior fins not connected to anterior fins, beginning in front of caudal septum and both anterior fins and posterior fins completely rayed (Fig. 20D, E). Caudal fin fully rayed and roughly round or triangle-shaped (Fig. 20C). Intestinal diverticula present (Figs 7A, 20B). Seminal vesicles longitudinally elongated along body (Fig. 20C). Eggs reaching posterior of ventral ganglion.

##### Distribution.

This species is found in the epipelagic (0–200 m depth) and mesopelagic zones (200–500 m depth) of Pacific and Indian Oceans (Pierrot-Bults and Nair 1991) and the epipelagic zone (0–100 m depth) of the Tosa Bay in Japan (Ohnishi et al. 2014). In this study, it was found in the epipelagic zone (0–120 m depth) of the Korea Strait and northern East China Sea (Fig. 1, stations KS06, nECS01 and nECS02).

##### Ecology.

This species is considered a warm-water Indo-Pacific indicator species because many individuals appear in high-temperature and high-salinity water and the distribution range is limited to the areas affected by warm currents (Park 1970).

##### Remarks.

Amongst the Korean specimens, the collarette was differently inflated, thick and covered the body surface. However, the position and shape of the corona ciliata and fins were consistent with the original description (Aida 1897). *Aidanosagittaregularis* is similar to *A.bedfordii*; however, these two species can be distinguished by the morphological difference in the eye pigments (elongated vs. roundish). We observed two specimens for CBE staining pattern: dorsomedian line, 28 dots; dorsolateral line, 34 dots; lateral line, 44 dots; receptors on the lateral fin, 3 dots; anterolateral receptors on the caudal fin, 2 dots; posterior receptors on the caudal fin, not observed due to damage.

#### ﻿Genus Sagitta

Taxon classificationAnimalia AphragmophoraSagittidae

Quoy & Gaimard, 1827

792BA0F1-B183-55FB-9E27-DE17465C1FF1

##### Diagnosis.

Rigid and opaque body. Collarette almost absent. Intestinal diverticula absent or present. Grasping spines not serrated. Two pairs of lateral fins completely rayed.

#### ﻿ Sagitta bipunctata

Taxon classificationAnimalia AphragmophoraSagittidae

Quoy & Gaimard, 1827

FA6C6113-13AD-571C-8989-B2B65A15F62B

Sagitta bipunctata : Quoy & Gaimard, 1827: 232–233 p., figs 1, 2, 6, 7; Aida 1897: 13–14 p., fig. 1; Ritter-Záhony 1908: 15 p., figs 2, 2A; Ritter-Záhony 1910: 2 p., Fowler 1906: 68 p.; Sund 1961: 110 p., table 1; Alvariño 1967: 44–49 p., fig. 26A–D; Dallot and Ducret 1969: 19 p., table 1; Srinivasan 1979: 8–9 p., fig. 3A–F; Michel 1984: 17–18 p., fig. 18; McLelland 1989: 163–164 p., figs 9A, 12I; Villenas and Palma 2006: 105 p., table 1

##### Material examined.

Yellow sea (33°0.111'N, 125°29.581'E), 0 – 86 m depth, oblique towing, July 2020.

##### Description.

Small, rigid and opaque body. Small head. Collarette almost absent (thin on neck). Eyes square-shaped with no eye pigments observed in this study. Intestinal diverticula absent. Forward end of anterior fins not visible. Posterior fins beginning in front of caudal septum and closing to seminal vesicles, completely rayed. Seminal vesicles elongated with small indentations and touching both lateral and caudal fins.

##### Distribution.

This species is found in the epipelagic (0–200 m depth) and mesopelagic zones (200–500 m depth) of Pacific, Indian and Atlantic Oceans (Pierrot-Bults and Nair 1991). This species appears in most of the seas across Korean waters.

##### Ecology.

*Sagittabipunctata* is known as a cosmopolitan species, appearing in temperate and tropical seas; it is an indicator species of high salinity and the presence of oceanic water (Pierce 1953). The temperature range in sampling stations of this study was 15.08–22.17 °C and the salinity range was 31.77–34.01 psu.

##### Remarks.

Only one immature individual was collected in this study. *Sagittabipunctata* can be distinguished from other Korean species by the following characteristics: absence of intestinal diverticular, presence of completely rayed lateral fins and the restricted position of collarette on the posterior part of the body. The seminal vesicles of Korean specimen were not sufficiently mature compared to the description of Alvariño (1967).

#### ﻿ Zonosagitta

Taxon classificationAnimalia AphragmophoraSagittidae

Tokioka, 1965a

F86D9852-A0D9-5F84-96B7-5ABC82CE5948

##### Diagnosis.

Rigid or flaccid and transparent or opaque body. Collarette small (almost absent). Intestinal diverticula absent. Grasping spines not serrated. Two pairs of lateral fins partially rayed. Anterior part of anterior fins elongated and rayless. Anterior fins longer than posterior fins.

### ﻿Key to species of *Zonosagitta*

**Table:** 

1	The anterior fins beginning at the middle of ventral ganglion, the seminal vesicles oval shaped touching neither the posterior fins nor caudal fin, small eyes with star-shaped eye pigments	***Z.bedoti***
–	The anterior fins beginning at the end of ventral ganglion, the seminal vesicles conical-shaped and touching both lateral fins and caudal fin, small eyes with “E” shaped eye pigments	***Z.nagae***
–	The anterior fins beginning at the middle of ventral ganglion, the seminal vesicles elongated with a roundish anterior protruding part, small eyes with star shaped eye pigments	***Z.pulchra***

#### ﻿ Zonosagitta bedoti

Taxon classificationAnimalia AphragmophoraSagittidae

(Béraneck, 1895)

8A9903D6-0442-5897-87A9-3084A22E7169

Figs 7D , 21A–E

Sagitta bedoti f. minor : Tokioka, 1959: 89–90 p., table 18.
Sagitta bedoti : Béraneck, 1895: 147–152 p.; Fowler 1906: 6–8 p., figs 1–8; Michael 1919: 255–257 p., figs 6, 20, 24, 30; Burfield and Harvey 1926: 94–95p., figs 1–2; Thomson 1947: 18 p.; Kitou 1966: 239 p., table 1; Alvariño 1967: 53–55 p., fig. 32A–D; Francisco 1977: 229–231 p., plate 2; Srinivasan 1979: 6–7 p., fig. 2A–G.

##### Material examined.

Korea Strait (33°0.000'N, 125°18.000'E), 0–75 m depth, oblique towing with conical net, Feb 2020, NIBRIV0000895300 (one specimen); northern East China Sea (31°30.000'N, 126°28.998'E), 0–82 m depth, oblique towing with conical net, Feb 2020 (one specimen).

##### Description.

**Figure 21.**
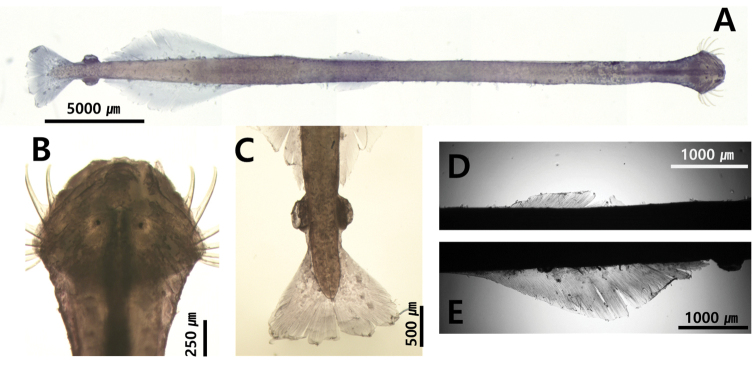
**A***Zonosagittabedoti* (dorsal view) **B** head **C** tail **D** anterior fin **E** posterior fin. Abbreviations: AF = anterior fin; E = eye; PF = posterior fin; SV = seminal vesicle.

Total body length ranged from 16–17 mm. Tail 3.71% of body length. Hooks 7. Anterior teeth 27–28 and posterior teeth 30–35, respectively. Opaque and rigid body (Fig. 21). Head wider than body (Figs 7D, 21A). Short and dense teeth. Intestinal diverticula absent (Figs 7D, 21B). “D”-shaped eyes with star-shaped eye pigments (Fig. 21B). Corona ciliata beginning behind eyes and elongated over neck (Fig. 21B). Anterior fins 26.5% of body length. Anterior fins beginning at middle of ventral ganglion, anterior of anterior fins narrow with rayless zone and posterior of anterior fins partially rayed. Anterior fins 1.3 times longer than posterior fins. Starting points of anterior fins 34.3% and ending points of anterior fins 60.0% of body length, respectively (Fig. 21A, D). Posterior fins 19.8% of body length. Starting points of posterior fins 63.7% and ending points of posterior fins 89.7% of body length, respectively. Posterior fins connected with anterior fins (Fig. 21A). Caudal fin triangular-shaped and completely rayed (Fig. 21C). Seminal vesicles spherical in shape with lateral rupture in mature specimen (Fig. 21C).

##### Distribution.

This species is found in the epipelagic zone (0–200 m depth) of the Pacific and Indian Oceans (Pierrot-Bults and Nair 1991) and the Tosa Bay in Japan (Ohnishi et al. 2014). In this study, it was found in the epipelagic zone (0–100 m depth) of the Korea Strait and northern East China Sea (Fig. 1: stations KS09 and nECS06).

##### Ecology.

*Zonosagittabedoti* is used as an indicator species in the front area where warm and cold water meet (Park 1970). The temperature range in the sampling location in this study was between 14.62 and 15.01 °C and the salinity range was 33.67–33.81 psu.

##### Remarks.

*Zonosagittabedoti* and *Z.nagae* are similar in morphology at the immature stage. However, because adult *Z.nagae* are relatively larger, immature *Z.nagae* may be misidentified as *Z.bedoti*. Adults of both species can be distinguished from each other by the shape of seminal vesicles and eye pigments. *Zonosagittanagae* has an “E”-shaped eye pigment, while *Z.bedoti* has a star-shaped eye pigment. The spot pattern on the body surface found through CBE staining is as follows: irregular spot pattern which continued from the head to the ventral ganglion and six spots along the outside of the body were symmetrical around the tail.

#### ﻿ Zonosagitta nagae

Taxon classificationAnimalia AphragmophoraSagittidae

(Alvariño, 1967)

9FBBEC20-39F8-5FF2-8B03-35E1894DA3CF

**Figure 22.**
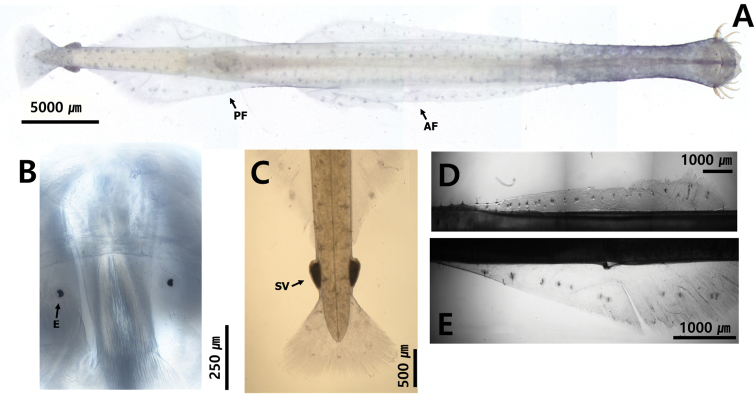
**A***Zonosagittanagae* (dorsal view) **B** head **C** tail **D** anterior fin **E** posterior fin. Abbreviations: AF = anterior fin; E = eye; PF = posterior fin; SV = seminal vesicle.

Figs 6D , 7C , 22A–E

Sagitta nagae : Alvariño, 1967: 55–58 p., fig. 34A–D.

##### Material examined.

Korea Strait (33°29.662'N, 125°30.881'E), oblique towing with MOCNESS, 32–58 m depth, July 2020, NIBRIV0000895297 (two specimens); Korea Strait (33°24.504'N, 127°54.600'E), 0–50 m depth, oblique towing with conical net, May 2019, two specimens; northern East China Sea (31°30.000'N, 125°17.100'E), 0–50 m depth, oblique towing with conical net, Feb 2020, NIBRIV0000895296 (one specimen).

##### Description.

Total body length ranged from 11–15 mm. Hooks 6–8. Anterior teeth 11–15 and posterior teeth 15–35, respectively. Rigid and opaque body (Fig. 22A). Long and dense teeth. Collarette present on neck (Fig. 22B). Intestinal diverticula absent (Fig. 7C). Eyes “D”-shaped with “E”-shaped eye pigments (Fig. 22B). Anterior fins 27.7% of body length and 1.5 times longer than posterior fins. Anterior fins beginning in front of ventral ganglion and partially rayed. Starting points of anterior fins 28.6% and ending points of anterior fins 59.8% of body length, respectively (Fig. 22A, D). Posterior fins 18.0% of body length. Starting points of posterior fins 67.8% and ending points of posterior fins 91.9% of body length, respectively. Posterior fins connecting with anterior fins partially rayed (Fig. 22A, E). Seminal vesicles conical-shaped with small indentations and well separated from posterior fins (Fig. 22C). Eggs reached posterior of anterior fins. Corona ciliata beginning behind eyes and elongated over neck (Fig. 22C).

##### Distribution.

This species is found in the epipelagic zone (0–200 m depth) of the Pacific and Indian Oceans (Pierrot-Bults and Nair 1991). In this study, the species was found in the epipelagic zone (0–120 m depth) of the northern East China Sea, the Korea Strait and the Yellow Sea (Fig. 1: stations KS03, KS07 and nECS05).

##### Ecology.

This species appears year-round in most of the seas around Korea, predominantly in the southern and western seas and shows high abundance in areas where warm and cold currents meet (Park 1970). In summer, the temperature range was 15.49–28.70 °C and the salinity was 30.92–34.01 psu. In winter, the temperature range was 11.47–11.58 °C and the salinity was 32.44–32.49 psu.

##### Remarks.

The anterior fin of *Zonosagittanagae* had a longer rayless zone than the posterior fin, clearly distinguishing it from *Z.bedoti*. CBE staining showed the following spot pattern on the body surface: an irregular spot pattern continued from the head to the middle of the ventral ganglion; symmetrical dots appeared near the rayless zone of each fin (13 spots on anterior fin, 6 spots on posterior fin).

#### ﻿ Zonosagitta pulchra

Taxon classificationAnimalia AphragmophoraSagittidae

(Doncaster, 1902)

90D4908F-19EC-5731-93B1-568DB28626F6

Sagitta pulchra : Doncaster, 1902: 213 p.; Fowler 1906: 72 p.; Michael 1919: 251–253 p., tables 7, 8; Burfield and Harvey 1926: 100 p., fig. 30; Thomson 1947: 19–20 p.; Kitou 1966: 239 p., table 1; Alvariño 1967: 34–35 p., figs 20–21; Francisco 1977: 231–233 p.; Srinivasan 1979: 29–30 p., fig. 16.

##### Material examined.

Korea Strait (34°17.868'N, 128°15.854'E), 0–60 m depth, oblique towing with MOCNESS, Feb 2020 (one specimen).

##### Description.

Rigid and transparent body. Collarette present. Intestinal diverticula absent. Grasping spine gently curved. Two rows of stout teeth arranged in comb shape. Two pairs of lateral fins partially rayed, anterior fins begin at the end of ventral ganglion. Seminal vesicles elongated with an anterior protruding part usually roughly round and touching both lateral fins and caudal fin.

##### Distribution.

The species is found in the epipelagic zone (0–200 m depth) of the Pacific and Indian Oceans (Pierrot-Bults and Nair 1991) and off the coast of Philippines (Noblezada and Campos 2008). In this study, it was found in the epipelagic zone (0–120 m depth) of the northern East China Sea, the Korea Strait and the Yellow Sea (Fig. 1: station KS02).

##### Ecology.

*Z.pulchra* is considered to be a neritic species (Bieri 1959; Pierrot-Bults and van der Spoel 2003). The temperature range in the sampling stations of this study was 11.95–14.79 °C and the salinity range was 33.59–34.00 psu.

##### Remarks.

Only one immature individual was collected in this study. The examined Korean specimen belongs to the genus *Zonosagitta* by the absence of intestinal diverticular and the presence of rayless zone in the anterior part of anterior fins. This Korean specimen was identified as *Z.pulchra* because its anterior fins were more angled than those of *Z.bedoti* or *Z.nagae*. The seminal vesicles of Korean specimen were not sufficiently mature compared to description of Alvariño (1967).

## ﻿Discussion

Based on a taxonomic review of newly obtained specimens from Korea, we confirmed the appearance of chaetognath taxa in Korean waters corresponding to one order, three families, 11 genera and 18 species. Taxonomically identified voucher specimens (*Krohnittasubtilis*, *K.pacifica*, *Pterosagittadraco*, *Aidanosagittacrassa*, *A.neglecta*, *A.regularis*, *Ferosagittaferox*, *F.robusta*, *Flaccisagittaenflata*, *F.hexaptera*, *Mesosagittaminima*, *Parasagittaelegans*, *Pseudosagittalyra*, *Sagittabipunctata*, *Serratosagittapacifica*, *Zonosagittabedoti*, *Z.nagae* and *Z.pulchra*) were obtained for the first time in Korea and their taxonomic and ecological features were reported. Although the overall morphological characteristics of the six species (*A.neglecta*, *F.ferox*, *K.pacifica*, *M.minima*, *S.bipunctata* and *Z.pulchra*) were mostly consistent with the previously-reported species, their essential characteristics have been briefly described in this study because only immature individuals of these six species were collected. On the contrary, the three species (*Decipisagittadecipiens*, *Serratosagittaserratodentata*, *S.pseudoserratodentata*) mentioned in literature have very poor taxonomic basis for their academic report (drawings, descriptions and voucher specimens), which can result in possible misidentification and their presence in Korean waters is questionable. Most of the samples in this study were collected in summer and winter. Extension of the investigation period to spring and autumn in future studies can facilitate identification of adult specimens of the above-mentioned six species or clarification of the presence or absence of the three suspicious species.

The detailed characteristics of the corona ciliata and fins of chaetognaths are important as taxonomic keys to distinguish genera, but they are difficult to observe under a stereomicroscope. In order to address this problem, we stained the surface of the specimens with CBE, which has rarely been used in the past for observing chaetognaths. The CBE staining of the Korean specimens clearly revealed the features of the corona ciliata, fins and body surface. The corona ciliata of *Aidanosagitta* is located from behind the eyes to the anterior part of the trunk, whereas that of *Sagitta*, *Serratosagitta*, *Ferosagitta*, *Parasagitta* and *Zonosagitta* extends from the front of the eyes to the neck; hence, the two groups could be clearly distinguished. *Flaccisagitta* is also distinctly differentiable from other genera as the corona ciliata extends from the front of the eyes to the anterior part of the neck. We propose the location and shape of these corona ciliata as additional taxonomic keys to distinguish Korean taxa at the genus level. On the contrary, *Krohnittasubtilis* had a circular corona ciliata located in front of the eyes, unlike other congeneric species, in which a circular corona ciliata appeared behind the eyes. Bieri (1974) has suggested the artificial position change of the corona ciliata due to damaged specimens. Therefore, the differences in corona ciliata between *Krohnittasubtilis* found in Korea and other known congeneric species need to be confirmed by examining more specimens.

The distribution of dots on the body surface was easily confirmed through microscopic observation after CBE staining. The dots pattern is expressed by a regular arrangement of tactile cilia distributed on the body surface of the chaetognaths (Aida 1897). The tactile cilia are reported to have two types of hair (Aida 1897; Feigenbaum 1978). One is transversally orientated ciliary fence organs and the other is longitudinally orientated ciliary tuft organs (Aida 1897; Bone and Pulsford 1978; Müller et al. 2014). The tactile cilia are capable of responding to water movement on the surface of the body or of detecting prey (Horridge and Boulton 1967). Recent studies have established the role of tactile cilia as nerve receptors (Müller et al. 2014). In this study, *Aidanosagitta*, *Serratosagitta* and *Ferosagitta* have similar round dots of symmetry based on the dorsal median line from the head to the tail, but their size and location are different for each genus. *Zonosagittanagae* and *Z.bedoti* also showed a marked difference from other genera by showing irregular spot patterns from the head to the ventral ganglion.

On the contrary, *Flaccisagitta* did not have similar spot patterns on the body surface between the two Korean species. Irregular spots on the body surface of the *Flaccisagittaenflata* were observed; however, *F.hexaptera* did not exhibit any spot patterns, similar to *Pseudosagittalyra*. Since the fully-grown body of this species is usually large with a size of 50 mm and more, it is presumed that the relatively thick epidermis prevented effective staining. The regularity of these patterns has also been observed in species such as *Sagittahispida*, *S.enflata*, *S.elegans*, *Spadellaschizoptera* and *Spadellacephaloptera* (Feigenbaum, 1977); however, evidence for establishing a connection with genealogy is lacking. Moreover, basic data obtained from a complete individual pattern will be valuable to explain the commonalities at genus levels. Further application of the CBE staining method to other taxa of Aphragmophora will clarify whether new features, such as the location and shape of the corona ciliata and the spot patterns on the body surface, are effective as genus or species grouping features.

## Supplementary Material

XML Treatment for Aphragmophora

XML Treatment for Krohnittidae

XML Treatment for Krohnitta

XML Treatment for Krohnitta subtilis

XML Treatment for Krohnitta pacifica

XML Treatment for Pterosagittidae

XML Treatment for Pterosagitta draco

XML Treatment for Sagittidae

XML Treatment for Serratosagitta

XML Treatment for Serratosagitta pacifica

XML Treatment for Mesosagitta

XML Treatment for Mesosagitta minima

XML Treatment for Flaccisagitta

XML Treatment for Flaccisagitta enflata

XML Treatment for Flaccisagitta hexaptera

XML Treatment for Pseudosagitta

XML Treatment for Pseudosagitta lyra

XML Treatment for Parasagitta

XML Treatment for Parasagitta elegans

XML Treatment for Ferosagitta

XML Treatment for Ferosagitta ferox

XML Treatment for Ferosagitta robusta

XML Treatment for Aidanosagitta

XML Treatment for Aidanosagitta neglecta

XML Treatment for Aidanosagitta crassa

XML Treatment for Aidanosagitta regularis

XML Treatment for Sagitta

XML Treatment for Sagitta bipunctata

XML Treatment for Zonosagitta

XML Treatment for Zonosagitta bedoti

XML Treatment for Zonosagitta nagae

XML Treatment for Zonosagitta pulchra
